# Phosphine-Functionalized Squaramides as Responsive Organogelators: Structure, Gelation, and Metal-Ion Sensing

**DOI:** 10.1021/acs.joc.6c00455

**Published:** 2026-07-14

**Authors:** Daniel Salvador-Gil, Sara Illescas-Lopez, Raquel P. Herrera, Federico Movilla, David Díaz Díaz, Modesto T. López-López, M. Concepción Gimeno, Araceli G. Campaña, Luis Álvarez de Cienfuegos

**Affiliations:** † Universidad de Granada (UGR), Departamento de Química Orgánica, C. U. Fuentenueva, Avda. Severo Ochoa s/n, Granada E-18071 Spain; ‡ Unidad de Excelencia Química Aplicada a Biomedicina y Medioambiente (UEQ), 16741 Universidad de Granada, Granada E-18071, Spain; § Departamento de Química Inorgánica, Instituto de Síntesis Química y Catálisis Homogénea (ISQCH), CSIC-Universidad de Zaragoza, Pedro Cerbuna 12, Zaragoza 50009, Spain; ∥ Center for Cooperative Research in Biomaterials (CIC biomaGUNE), 90216 Basque Research and Technology Alliance (BRTA), Paseo de Miramón 194, Donostia-San Sebastián 20014, Spain; ⊥ Laboratorio de Organocatálisis Asimétrica. Departamento de Química Orgánica, Instituto de Síntesis Química y Catálisis Homogénea (ISQCH), CSIC-Universidad de Zaragoza, Pedro Cerbuna 12, Zaragoza 50009, Spain; # Nanoscopy-UGR Laboratory, Faculty of Pharmacy, University of Granada, Granada E-18071, Spain; ∇ Departamento de Química Orgánica, Universidad de La Laguna, Avda. Astrofísico Francisco Sánchez 3, La Laguna, Tenerife 38206, Spain; ○ Instituto Universitario de Bio-Orgánica Antonio González, Universidad de La Laguna, Avda. Astrofísico Francisco Sánchez 2, La Laguna, Tenerife 38206, Spain; ◆ Universidad de Granada (UGR), Departamento de Física Aplicada and Research Unit “Modeling Nature” (MNat), C. U. Fuentenueva, Avda. Severo Ochoa s/n, Granada E-18071, Spain; ¶ Instituto de Investigación Biosanitaria ibs.GRANADA, Av. De Madrid 15, Granada 18016, Spain

## Abstract

Phosphine-functionalized squaramides represent a promising class of low-molecular-weight gelators (LMWGs) capable of forming supramolecular organogels in a variety of organic solvents, including alcohols. In this work, a series of phosphine-containing squaramides was designed and evaluated, revealing that subtle structural differences critically influence gelation ability. Molecular dynamics simulations demonstrate that the phosphine functionality plays a key role in directing self-assembly, promoting the formation of fibrillar networks responsible for gel stabilization. The resulting organogels exhibit a selective response toward metal ions, with gold precursors inducing rapid and complete gel collapse even at low concentrations, while other cations produce only minor or no macroscopic effects. Experimental observations, supported by molecular dynamics simulations, indicate that Au^3+^ cations strongly interact with the phosphine moieties, promoting a reorganization of the supramolecular aggregates into more compact structures that disrupt the percolating fibrillar network. This coordination-driven restructuring highlights the sensitivity of phosphine-containing supramolecular assemblies to specific metal–ligand interactions. These findings demonstrate how the incorporation of metal-binding sites into squaramide-based gelators enables the development of responsive soft materials whose structure and stability can be selectively modulated by external chemical stimuli.

## Graphical abstract

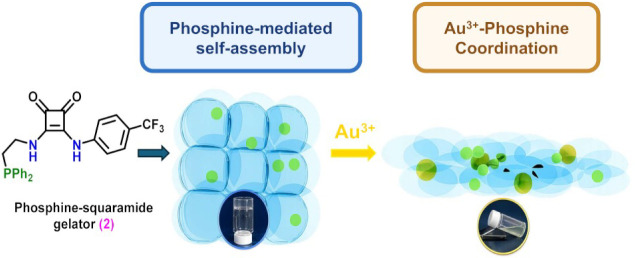

## 1 Introduction

Squaramides have gained considerable importance in various fields of chemistry and biomedicine.^1–3^ Their distinctive structural configuration, consisting of a cyclobutenedione ring with two amine units, endows them with a strong ability to form hydrogen bonds and to act as acceptor, donor, or dual donor–acceptor groups. These features are fundamental in molecular recognition and asymmetric catalysis.^1–6^ Furthermore, their structural rigidity and the ease with which chiral derivatives can be prepared make them versatile and valuable building blocks.^1–5^ Recent studies have demonstrated the potential of squaramides as building blocks for supramolecular self-assembly.^2,6–9^ The strong directionality and stability of squaramide aggregates have promoted their use as low-molecular-weight gelators (LMWGs)^6,10,11^ capable of forming organogels^1,6,10^ and hydrogels^2,10,12^ with fibrillar or nanofiber morphologies.^1,10–14^ Alegre-Requena et al. reported the organogelation ability of several squaramide derivatives, including a squaramide diester and its diacid precursor, finding that the diester exhibited superior gelation ability across a broad range of solvents.^1^ The nature of the solvent, the concentration, and the structure of the gelator all exert a significant influence on the properties of the resulting gels.^1,2,6,15^ For instance, chiral *N*,*N*′-disubstituted squaramides have been shown to form nanostructured alcogels that respond to multiple stimuli across a range of alcoholic solvents, even at low concentrations.^6,16^

By combining their ability to form gels with their capacity to coordinate metal ions,^14,17^ squaramides can also form metallogels. These gels, which incorporate metal ions into their supramolecular network,^15,18,19^ offer tunable properties, including luminescent, redox, and magnetic responses.^15,18^ The formation of metallogels from squaramide complexes with Cu^2+^ ions has been explored,^8,18,19^ and the results indicate that gelation depends on the nature of the anions and on the cooperativity between metal coordination and multiple hydrogen bonds.^6,8,15,18,20^ It has been shown that certain metal ions can induce collapse of the gel network, thereby underscoring the importance of ion selectivity within these systems.^6,8,15,18,21^

Despite extensive research on squaramides in self-assembly, catalysis, and ion detection, the functionalization of these molecules with phosphine ligands remains scarcely explored. Phosphine-squaramide conjugates reported so far have been used mainly as bifunctional ligands in silver-free autocatalysis.^22^ However, the integration of this phosphine-squaramide architecture, which is fundamental for metal coordination, into responsive soft-matter matrices such as organogels, and the exploration of its potential for metal-ion sensing outside catalytic contexts remain significant gaps in current knowledge.^22^ In this study, we investigated the self-assembly behavior of three phosphine-functionalized squaramide derivatives (**1**, **2**, and **3**) as well as three others (**4**, **5**, and **6**) not having a phosphine group as model compounds (Figure 1). Among them, **2** was found to be particularly effective, giving rise to self-supported organogels through a *heating–cooling* process assisted by ultrasound in methanol. After obtaining the organogel, a series of experiments were conducted to evaluate its stability and responsiveness to various cations, including Ag^+^, Na^+^, Ca^2+^, Cu^2+^, Au^3+^, Cr^3+^, Yb^3+^, and Eu^3+^. These experiments revealed that the addition of Au^3+^ induced collapse of the organogel.

**Figure 1:**
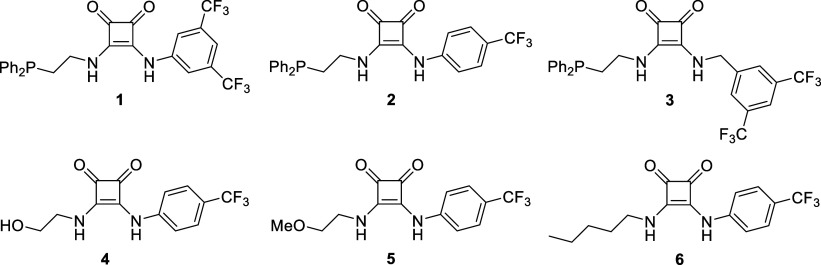
Molecular structures of functionalized squaramide derivatives containing a phosphine group (**1**, **2**, and **3**) and other functionalities (**4**, **5**, and **6**).

Overall, this work establishes phosphine-functionalized squaramides as a new class of responsive supramolecular organogelators, extending their functional scope beyond molecular recognition to metal-ion sensing. By coupling gelation with selective metal coordination, these systems open new opportunities for the design of adaptive soft materials with potential applications in environmental monitoring and advanced materials development.

## 2 Discussion of Results

### 2.1 Molecular Design of Squaramide Derivatives

Supramolecular gels rely on reversible noncovalent interactions that enable dynamic sol–gel transitions in response to external stimuli.^1,6,13,18^ In this context, squaramides emerge as attractive molecular platforms, as they combine strong and directional hydrogen-bonding capabilities with additional stabilizing interactions such as π–π stacking, arising from the partially aromatic character of the four-membered ring.^2,3^ In order to enhance both the hydrogen-bonding properties and the functional versatility of these systems, a series of squaramide derivatives bearing trifluoromethyl (CF_3_) substituents were designed.^2,10,23^ From a molecular design standpoint, CF_3_ groups act as strong electron-withdrawing substituents, increasing the acidity of the squaramide NH protons and thereby strengthening hydrogen-bonding interactions.^2,10,22,24^ This effect has been widely studied in the design of highly active hydrogen-bonding organocatalysts.^4,22^ To expand the functional scope of these squaramide derivatives, a second substituent capable of coordinating metal ions was introduced (compounds **1** to **3**, Figure 1). Specifically, a phosphine moiety was chosen as a suitable ligand for coordination to interesting metal atoms including gold, silver, and copper, which have been demonstrated to influence the biological and physicochemical properties of metal complexes. The incorporation of a phosphine group enables metal coordination and offers the possibility to tune properties such as solubility, cytotoxic activity, and stability through ligand modification.^10,22^ Beyond its role as a metal-binding site, the incorporation of a diarylphosphine unit was expected to influence the self-assembly behavior by introducing additional directional and polarizable interactions, potentially affecting supramolecular aggregation. Besides, squaramides **4**–**6** (Figure 1) were synthesized as model compounds bearing a CF_3_ electron-withdrawing substituent but without a coordinating phosphine moiety. Instead, hydroxy, methoxy, or alkyl groups were introduced in **4**, **5**, and **6**, respectively. The targeted functionalized squaramides were synthesized using a straightforward one-pot procedure in methanol.^25^ In the first step, an aniline derivative bearing CF_3_ substituents was introduced, followed by the addition of the functionalized-containing fragment. Synthetic procedures as well as characterization compounds are provided in the Experimental Section.

### 2.2 Self-Assembly Behavior and Gelation Studies

The self-assembly behavior of **1**, **2**, and **3** was first evaluated through systematic gelation tests in a range of organic solvents using a standard heating–cooling protocol. In this method, the compound was dissolved upon heating (Figure 2a) and subsequently allowed to cool under resting conditions.^1,13^ For the present systems, a slight modification of this procedure was introduced, as it was experimentally observed that, after heating, lower gelation concentrations could be achieved when the samples were first subjected to ultrasound for 10 min and then allowed to stand at room temperature.^1,6,8^ Gelation was initially screened in four representative solvents (methanol, acetonitrile, chloroform, and toluene), and the formation of a gel was confirmed by the absence of free-flowing solvent at the surface of the vial, as assessed by the vial inversion test. Compound **1** bearing two CF_3_ groups was found to be insoluble in acetonitrile, toluene, and chloroform and only exhibited gelation behavior in methanol at a relatively high concentration of 10 mg/mL. Although a slight tendency toward gel formation was observed in methanol, further experiments in different alcohols revealed that **1** predominantly formed solid aggregates encapsulating the solvent rather than well-defined supramolecular gels (see Table S1). This behavior was consistently observed in several primary alcohols, while precipitation occurred in 2-propanol. The application of an alternative heating–ultrasound protocol did not improve the gelation behavior of **1**, as precipitation was systematically observed under these conditions. Overall, these results indicate that this compound did not efficiently form extended supramolecular networks capable of immobilizing the solvent. The gelation behavior of **3**, which differs from **1** by the introduction of an additional methylene unit, was subsequently examined using the same set of solvents. In contrast to **1**, compound **3** being more flexible due to the presence of an additional methylene group showed a higher tendency to gel, forming gels in methanol, acetonitrile, and toluene, while remaining insoluble in chloroform. A critical gelation concentration (CGC) of 5 mg/mL was obtained in toluene and acetonitrile, whereas a higher concentration of 10 mg/mL was required in methanol. To further explore its gelation potential, **3** was tested in additional solvents, including ethyl acetate, nitromethane, DMSO, xylene, chlorobenzene, benzonitrile, and mesitylene. As summarized in Table S2, improved gelation was only observed in ethyl acetate and nitromethane, where the CGC could be reduced to 4 mg/mL. However, despite these improvements, the resulting materials exhibited optical appearances similar to those obtained for **1**, corresponding to aggregated solids rather than homogeneous gel networks. The heating–ultrasound protocol did not lead to a significant enhancement in the stability of these gels. In contrast to **1** and **3**, compound **2** bearing only one CF_3_ in the *para-*position displayed a markedly enhanced ability to form supramolecular gels. Using the heating–cooling protocol, **2** readily formed self-supported organogels in both methanol and acetonitrile (Figure 2b,c). Notably, a minimum CGC of 2 mg/mL was achieved in acetonitrile, representing the lowest gelation concentration observed among the squaramide derivatives investigated in this study. Gelation was also observed in nitromethane at a comparable concentration (Figure 2d), while no stable gels were formed in ethyl acetate or DMSO and suspensions were formed in chloroform (Figure 2e) and toluene (Figure 2f). Given the pronounced tendency of **2** to gel in polar protic solvents, its behavior in a series of alcohols was subsequently investigated. The compound formed stable alcogels in a wide range of alcohols, including primary, secondary, and tertiary alcohols, as well as both linear and branched systems (Table S3). The rheological properties of organogels of **2** formed in methanol and acetonitrile were analyzed under oscillatory shear strain (Figure 2g–i and Figures S1–S3). All samples displayed the characteristic behavior of weakly cross-linked supramolecular gels, characterized by approximately constant values of both G′ (storage modulus) and *G*″ (loss modulus), within the linear viscoelastic region (LVR), together with *G*′ values significantly higher than *G*″ at low shear strain amplitudes (γ_0_) (Figure 2g and Figures S1B and S2B).^26,27^ Comparative analysis of the viscoelastic moduli performed on the mean log-transformed *G*′ and *G*″ of independent replicates (Welch’s *t*-test) revealed no significant differences between the mechanical properties of the gels formed in methanol and acetonitrile within the LVR (*p* > 0.05) (Figure 2g,i and Figures S1 and S2), although gels formed in methanol exhibited slightly higher average *G*′ values (Figure 2i). In both cases, the organogels displayed weak-gel behavior, as indicated by tan­(δ) values higher than 0.1.^26,28,29^ Gelation kinetics, monitored through the evolution of *G*′ and *G*″ over time (Figure 2h and Figure S3), revealed a remarkably rapid gelation process, with both moduli reaching nearly constant values within the first 10 min in all replicates.^30^

**Figure 2:**
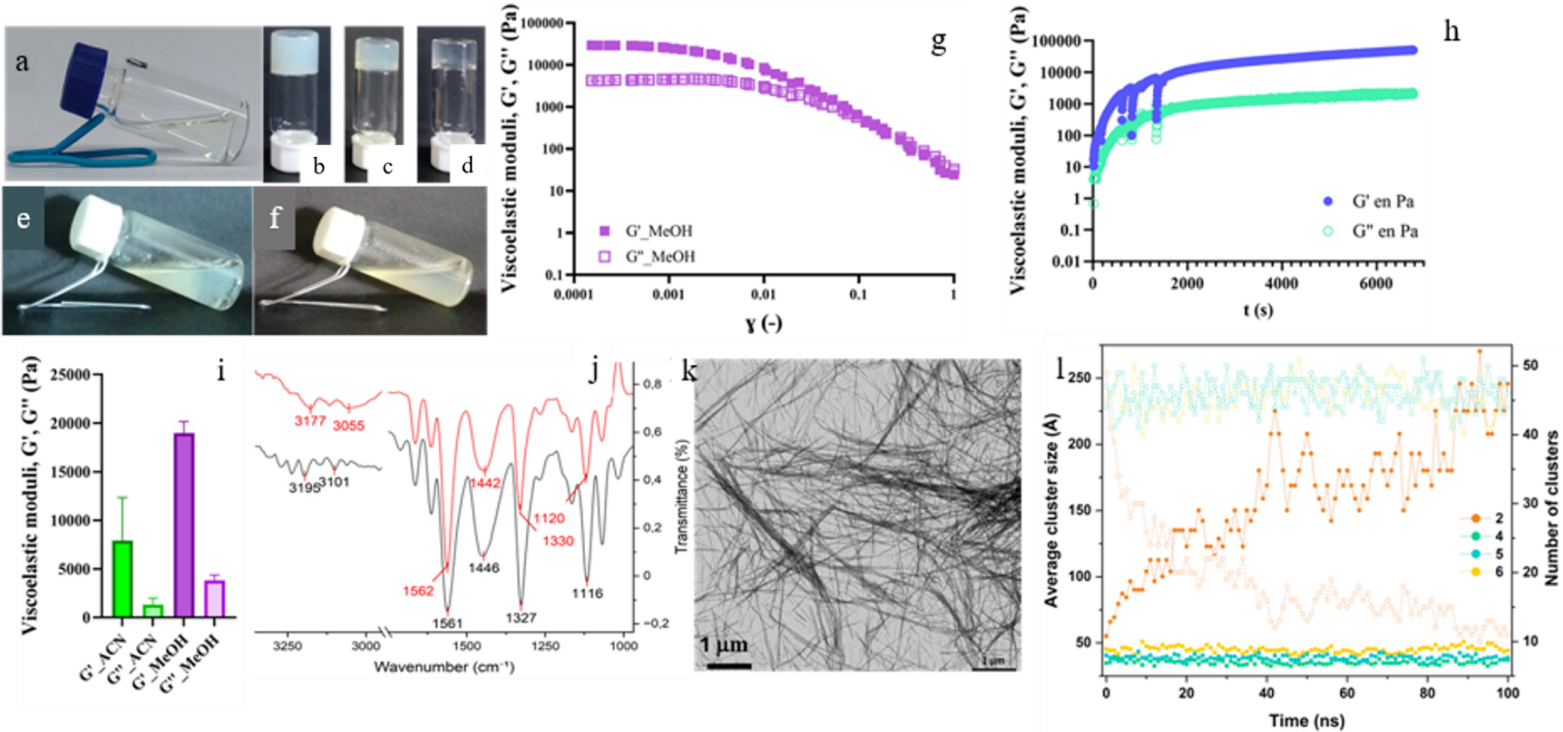
a) Macroscopic appearance of **2** methanol solution before ultrasound. Macroscopic appearance of gels of **2** formed in b) methanol (5 mg/mL), c) acetonitrile (5 mg/mL), and d) nitromethane (2 mg/mL). Suspensions of **2** formed in e) chloroform (5 mg/mL) and f) toluene (5 mg/mL). g) Viscoelastic moduli as a function of the shear strain amplitude in the oscillatory strain test at constant frequency (1 Hz) for organogel **2** (5 mg/mL) in methanol. h) Variation of the viscoelastic moduli (*G*′ and *G″*) during gel **2** formation (5 mg/mL) in methanol, i) comparative values of viscoelastic moduli (*G*′ and *G″*) of gels formed in acetonitrile (green) and methanol (purple), and j) ATR-IR spectra of compound **2** solid (black) and gel (red). k) Transmission electron microscopy image of **2** fibers formed in methanol. l) Average cluster size (filled circle) and number of clusters (empty circles) computed from the molecular dynamics simulation of methanol solutions for **2**, **4**, **5**, and **6**.

The ATR-FTIR spectra of the squaramide derivative **2** in the solid state, xerogel (Figure S6), and methanol gel revealed only marginal alterations (Figure 2j and Figures S4–S6). Minor frequency shifts (Δλ < 4 cm^–1^) were observed for characteristic peaks, including the 1560 cm^–1^ band, crucial in the squaramides, as it represents the delocalization of the system in the four-membered ring (C=O and C=C stretchings), 1440 cm^–1^ (δ CH_2_ “scissoring”), and 1330 cm^–1^ (aromatic C–N). This suggests that **2** exists in an aggregated state in the solid phase, driven by hydrogen bonding and π-stacking interactions, similar to those previously described for other squaramide organogelator analogues.^6^

Figure 2k shows a transmission electron microscopy (TEM) image of high-aspect-ratio fibers of **2** formed in methanol. As can be seen in the image, the fibers are micrometers in length and have diameters between 223 and 148 nm.

This broad solvent tolerance suggests that gelation is governed by a dominant intermolecular interaction and by a balanced hydrophilic–hydrophobic character that favors self-assembly in alcoholic media. The resulting alcogels exhibited remarkable stability, with lifetimes ranging from one to two months under ambient conditions. Gels formed in 1-pentanol and 2-butanol remained stable for up to four months without noticeable degradation or solvent release. The CGC values obtained for **2** varied depending on the solvent, ranging from 25 to 4 mg/mL. These values indicate that many solvent molecules are immobilized within the supramolecular network formed by the gelator. The highest solvent-to-gelator ratios were observed for nitromethane and acetonitrile, with CGC values of approximately 1.9 and 2 mg/mL, respectively. These results highlight the strong dependence of gelation behavior on subtle structural variations within the squaramide framework. While **1** and **3** exhibit limited or poorly defined gelation, **2** emerges as an efficient LMWG capable of forming stable and long-lived organogels and alcogels across a broad range of solvents. Notably, the markedly enhanced gelation ability of **2** cannot be explained solely based on hydrogen bonding interactions, since all three derivatives share the same squaramide core. To directly assess the role of the phosphine group in the self-assembly process, a series of structurally related squaramide derivatives lacking this functionality (**4**–**6**) were synthesized and investigated (Figure 1). Although it might initially be thought that compound **4**, due to the presence of polar groups such as OH, could interact intramolecularly through hydrogen bonding and thus form stable gels, the experimental results showed that under the same concentration and temperature conditions, compound **4** precipitated from the medium or formed a series of aggregates. The same results were also obtained for **5** and **6**, showing that none of these derivatives were able to form self-supported organogels proving that the phosphine group, under the conditions studied, was necessary for the self-assembly process. To rationalize these experimental observations at the structural level, molecular dynamics simulations were carried out. These simulations were designed to probe the early stages of aggregation and clustering in solutions, rather than to reproduce the gel phase itself. The systems studied were composed of methanol as the solvent, and the corresponding derivatives studied in this work (**2**, **4**, **5**, and **6**) were in a proportion that respects the exact same concentration (9.36 mM) employed for the gelification test (further details regarding molecular dynamics simulation parameters and workflow could be found in the Experimental Section). These simulations of some representative squaramides clearly showed the crucial impact of the diarylphosphine group on their self-assembly properties as their structures only differ in the final group, going from diphenylphosphine (**2**) to hydroxy, methoxy, or propyl groups (**4**, **5**, and **6**, respectively) (Figure 2l). This is particularly evident when analyzing the main supramolecular interactions developed in the system (Figure 3a and selected representative interaction in panels b, c, and d) and the clustering effect observed during self-assembly simulations. This later was done by comparing the average cluster size and number of clusters developed for each compound in methanol solutions. This analysis revealed that this compound develops a sharp increase in average cluster size alongside a proportional decrease in the number of clusters, a behavior consistent with the early stages of self-assembling systems. It is important to notice that this behavior was unique to **2**. Additionally, although multiple dynamic supramolecular interactions could be found during the whole simulation throughout the aggregates formed, all the observed conventional H-bonds developed arise from the interaction between the amine groups of the compounds and the solvent, with an almost negligible contribution to the aggregate internal noncovalent framework (Figure 3b). Nevertheless, it is interesting to point out that this lack of conventional H-bonds seems to be compensated by the ubiquitous presence of H-bonds of nonconventional nature (specifically, F···H–C and O···H–C, detailed in Figure 3c).^31^ Even more, specific interactions involving fluor-carbon pairs could also be observed (Figure 3d). Interestingly, in the case of these tetrel-like interactions, while these typically require the presence of atoms heavier than carbon to act as the tetrel center,^32^ the combination of the high electronegativity of oxygen and the extreme strain within the squaramide cycle seems to significantly activate the σ-hole, thus enhancing its affinity toward fluorine. On the other hand, although it could be expected that phosphorus atoms would be prone to develop pnictogen bonds with fluorine atoms due to their polarizability,^32^ the two surrounding aryl groups fail to provide sufficient electron density withdrawal to activate their σ-hole. Furthermore, these groups also impose significant steric hindrance, strongly inhibiting bonding capability. Consequently, the formation of a P–F pnictogen bond in this supramolecular aggregate appears elusive.

**Figure 3:**
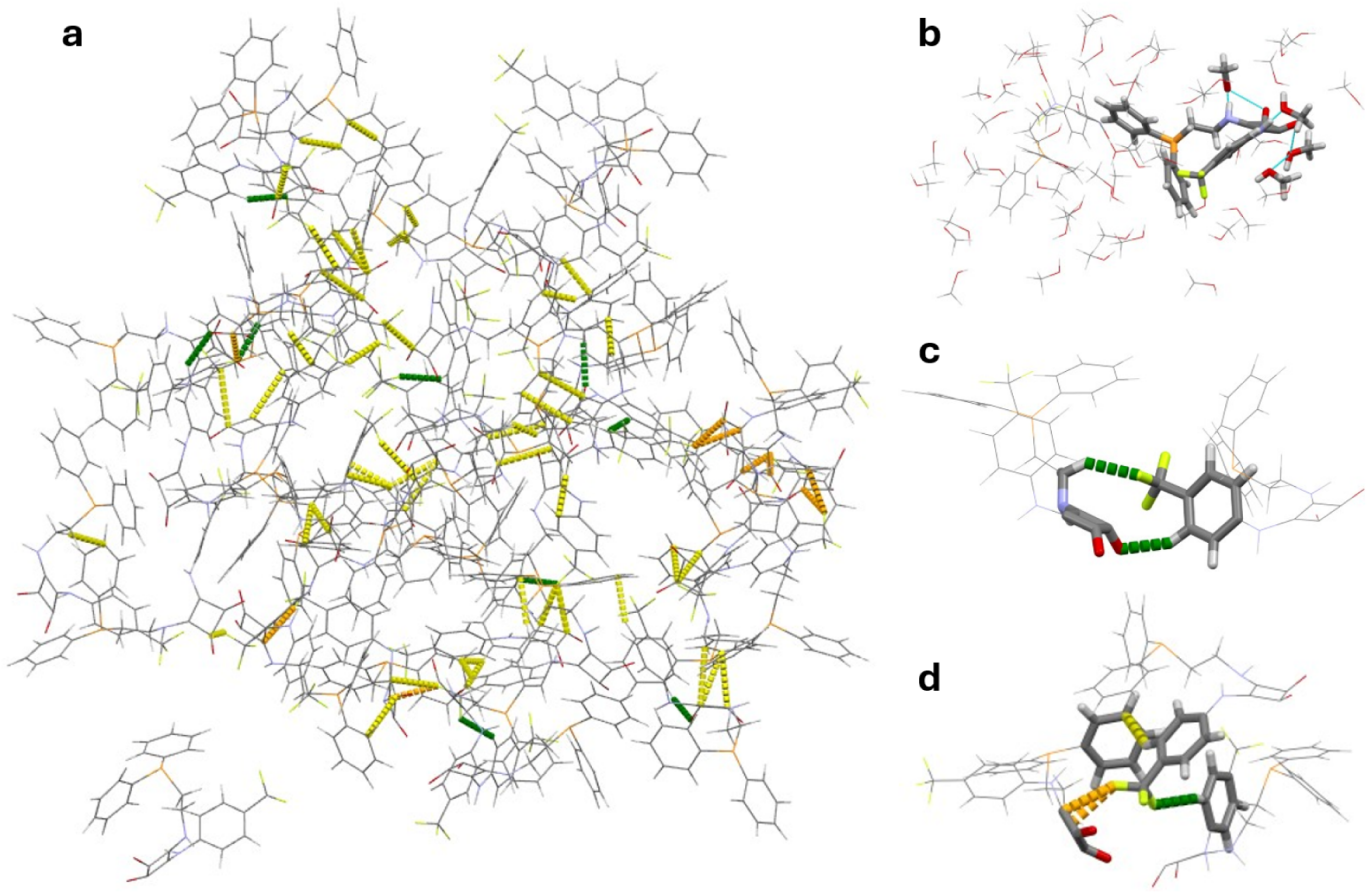
(a) Cluster generated by the self-assembly of derivative **2** in methanol during molecular dynamics simulations. Noncovalent interactions depicted throughout the supramolecular assembly are detailed in green for H-bonds, orange for F–C tetrel bonds, and yellow for dispersive interactions. (b) Detail of the solvent–derivative H-bond interactions. (c) and (d) are zoomed portions of the supramolecular arrangement where details of the present noncovalent interactions can be appreciated. Color code for the atoms: Carbon atoms are represented in gray, hydrogen in white, nitrogen in light blue, oxygen in red, phosphorus in orange, and fluor in yellow.

Nevertheless, it stands out that when compared to the structurally similar **4**, which has a strong hydrogen-bond donor group, no clustering was observed. This clearly indicates that the introduction of the diarylphosphine group, although not due to the addition of potentially active pnictogen centers, was still fundamental in designing a low-molecular-weight organogelator with particularly interesting self-assembly properties.

### 2.3 Metal-Ion Responsiveness and Selectivity

To evaluate the responsiveness of the supramolecular organogels formed by **2** toward metal cations, methanol was selected as a representative solvent as it allowed both robust gel formation and homogeneous metal-ion delivery. The behavior of the gel network was investigated in the presence of a series of metal ions with different valency numbers, including Ag^+^, Na^+^, Cu^2+^, Ca^2+^, Pd^2+^, Au^3+^, Eu^3+^, and Yb^3+^. Once the gel had formed, 100 μL of solutions of different metal salts were added individually to evaluate their effect on the gel network. In order to avoid introducing additional variables, chloride was used as the counterion in all cases, except for Ag^+^, which was added as AgNO_3_ due to the low solubility of AgCl. The effect of metal-ion concentration was systematically evaluated in the range of 0.025 to 0.1 equiv relative to the squaramide concentration (2.5 mg/mL). A markedly different response was observed depending on the nature of the cation. The addition of Au^3+^ resulted in complete gel collapse instantaneously at all concentrations tested, indicating a strong perturbation of the supramolecular network. This distinctive behavior likely arises from the unique chemical reactivity of gold species under the present conditions. In methanol, and in the presence of phosphine functional groups, Au^3+^ precursors are susceptible to chemical transformation, including partial reduction and the formation of lower-valent, highly coordinated, or multinuclear gold species. Phosphines are well-known to stabilize such species through strong donor interactions, and alcohols can also participate in redox processes or facilitate the formation of small gold aggregates. As a result, the gold present in the system may evolve into strongly coordinating centers or small clusters capable of simultaneously interacting with multiple phosphine moieties of compound **2**. These interactions can promote local densification, cross-linking, or structural reorganization of the supramolecular assemblies, ultimately disrupting the delicate balance of noncovalent forces required to sustain the fibrillar network and leading to macroscopic gel collapse.

In contrast, the presence of Na^+^, Ca^2+^, Pd^2+^, Eu^3+^, and Yb^3+^ did not induce any macroscopic changes in the gel structure, which remained intact across the entire concentration range investigated. For Ag^+^ and Cu^2+^, gel collapse was only observed at higher metal loadings (0.05, 0.075, and 0.1 equiv), while lower concentrations had no apparent effect on the gel integrity (Figure 4a–c). This pronounced cation-dependent behavior suggests that there must be a specific affinity toward gold ions that is not observed for other cations with similar charge-to-radius ratios and that metal coordination alone is not sufficient to significantly disrupt the supramolecular network. The final pH values of all gel samples after metal addition were found to be comparable, ruling out pH variations as the primary cause of the gel collapse. To gain further insight into the structural changes occurring within the gel network, TEM analysis was performed before and after metal addition (Figure 4d–f and Figures S7–S16). In the presence of Au^3+^, Pd^2+^, Yb^3+^, and Eu^3+^, a pronounced disruption of the fibrillar network was observed, characterized by the disappearance of extended fibers and the formation of droplet-like aggregates, accompanied by residual short fiber fragments. In the case of Pd^2+^, Yb^3+^, and Eu^3+^, these microscopic changes did not translate into macroscopic gel collapse, indicating that partial disruption of the fibrillar network was not sufficient to destabilize the bulk gel. On the contrary, in the presence of the gold precursor, a complete macroscopic collapse of the gel happened (Figure 4c). Moreover, the simultaneous presence of gold cations with any of the other metal ions investigated also led to gel collapse, underscoring the dominant role of gold in destabilizing the supramolecular assembly (Figures S17–S24).

**Figure 4:**
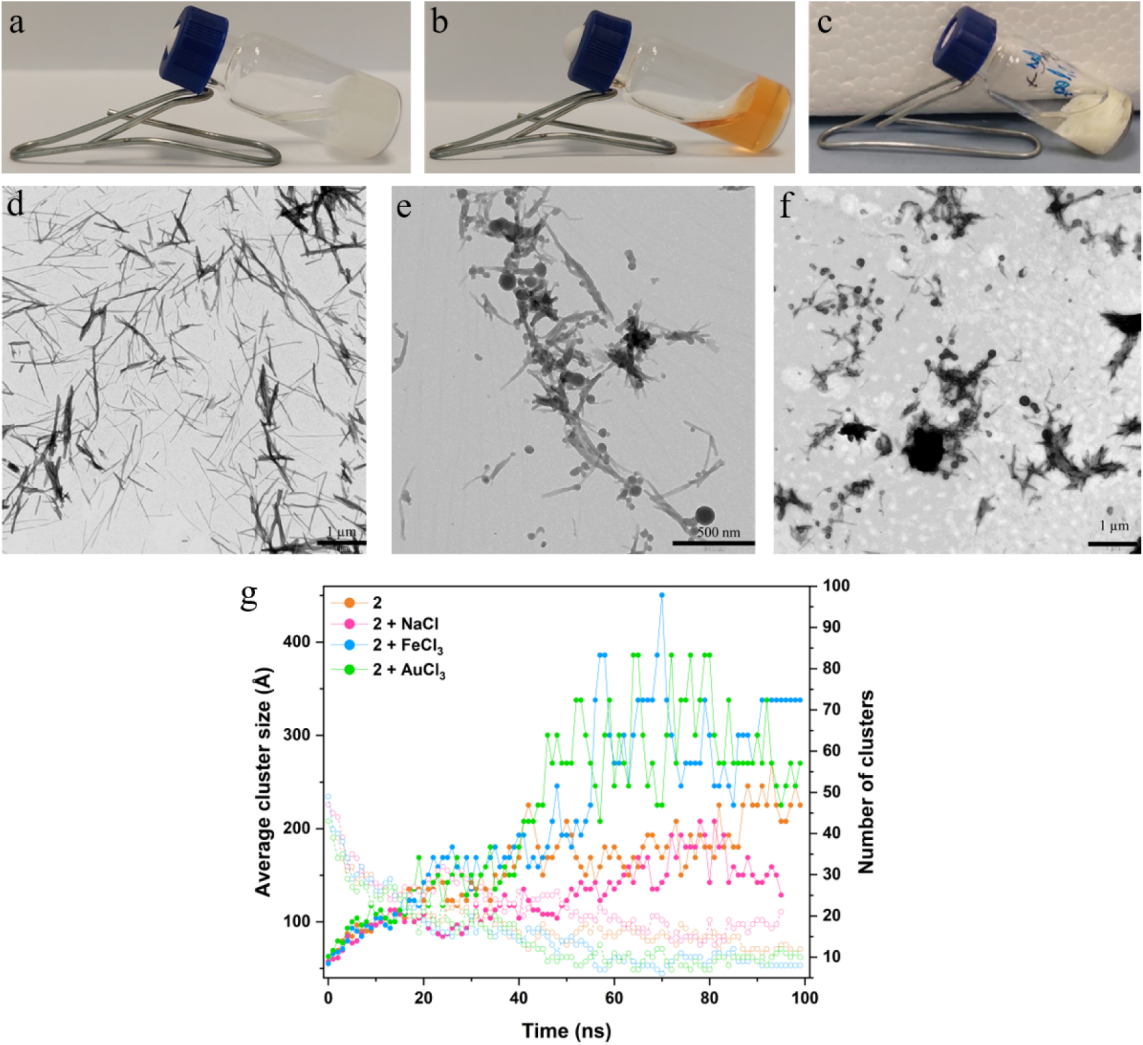
Macroscopic appearance of **2** organogel in methanol after the addition of salt solutions containing a) sodium chloride (0.22 mM), b) palladium chloride (0.22 mM), and c) tetrachloroauric acid (0.22 mM). Transmission electron microscopy image of **2** fibers after the addition of d) sodium salt, e) palladium salt, and f) gold salt. g) Average cluster size (filled circle) and number of clusters (empty circles) computed from the molecular dynamics simulation of **2** methanol solutions containing different chloride salts.

To assess the reversibility of the gold-induced gel collapse and to prove the specificity of the metal–gelator interaction, additional experiments were performed using cysteine as a competing chelating agent. Cysteine was selected due to its strong affinity for gold ions through thiol coordination.^33^ When cysteine was added during the gelation process, the gel samples exposed to the highest concentration of Au^3+^ collapsed within 24 h, whereas those treated with lower Au^3+^ concentrations largely preserved their macroscopic gel structure. TEM analysis revealed a significant reduction in the formation of droplet-like aggregates and a partial preservation of the fibrillar network under these conditions, supporting the hypothesis that cysteine acted as an effective gold scavenger and mitigated disruption of the supramolecular gel network (Figures S25–S26). These observations indicate that the selective response of the organogel toward gold originates from strong and specific interactions between gold species and the phosphine-containing squaramide scaffold. To prove P–Au interactions, ^31^P­{^1^H} NMR of the gel and after the addition of 0.1 eq of Au^3+^ was carried out (Figure S27). The ^31^P­{^1^H} NMR signal of the free phosphine, initially observed at −22.4 ppm in the gel state, almost completely disappeared upon addition of the gold­(III) salt. Concurrently, three main new resonances appeared at 20.7 (br), 33.5, and 77.4 ppm. The presence of multiple phosphorus signals suggests the formation of several gold–phosphine species. The higher-field resonances are likely associated with molecular Au–phosphine complexes in distinct coordination environments, whereas the downfield signal at 77.4 ppm may indicate the formation of a small phosphine-stabilized gold cluster. Overall, this behavior supports a dynamic coordination process in which a limited amount of gold interacts with multiple phosphine moieties, leading to a complex distribution of coordinated species. Such interactions can alter the supramolecular packing required for fiber growth and stabilization. Consistent with this interpretation, molecular dynamics simulations performed over the same system depicted before but including auric chloride as Cl^–^ and Au^3+^ ions in the same 1:3 ligand:cation proportion studied experimentally reveal that gold species promote the formation of larger and denser supramolecular clusters. These were characterized by an inward orientation of phosphine groups toward the coordinated metal centers (Figure 5a and detailed depiction on panels b, c, and d). This metal-induced reorganization contrasts with the soft, noncovalent bond-dominated packing observed in the absence of coordinating cations, providing a molecular-level rationale for the selective and irreversible gel collapse triggered by gold.

**Figure 5:**
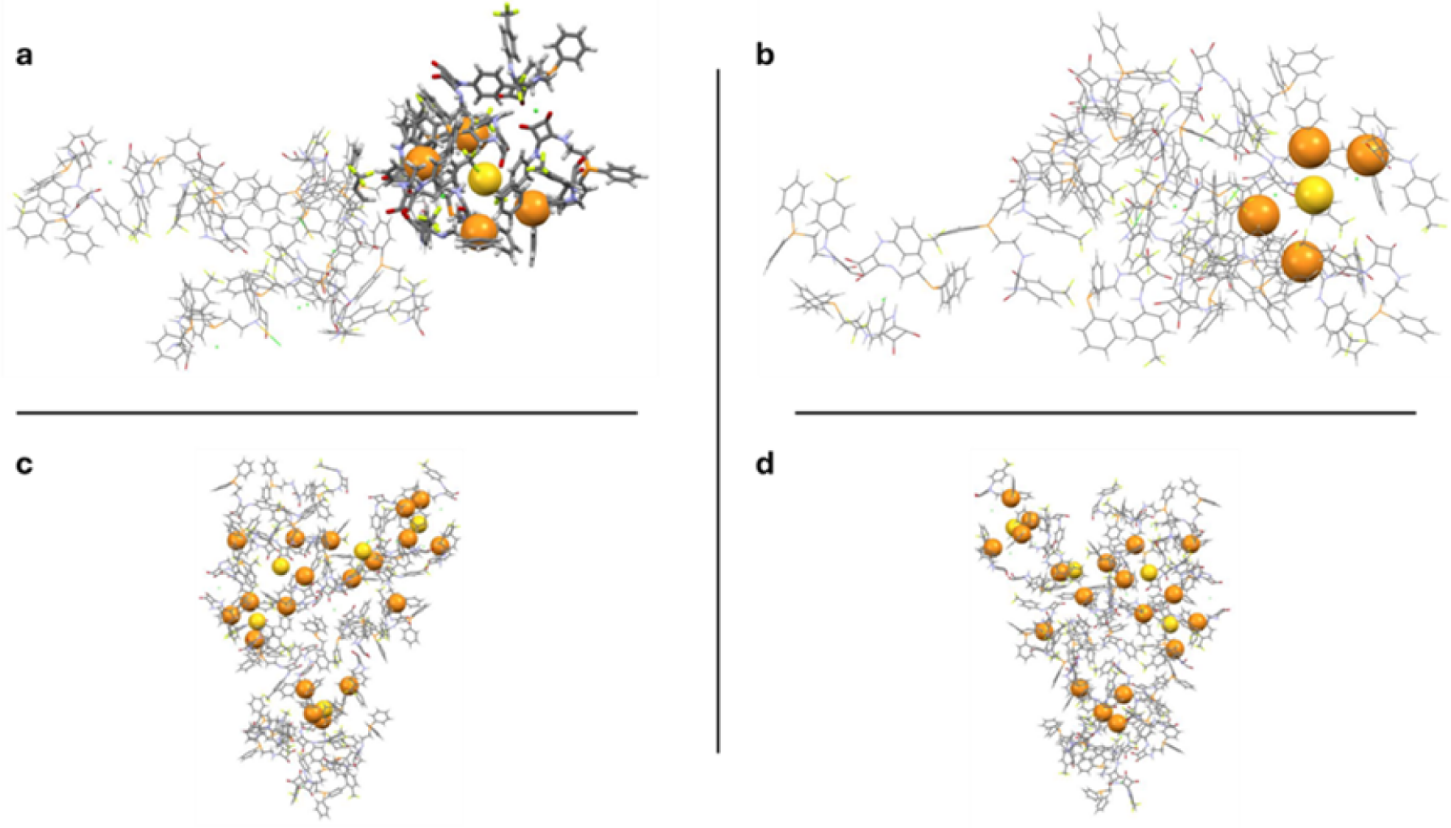
Selected frames observed during molecular dynamics simulations of the self-assembly of a **2** cluster in methanol in the presence of gold cations. Phosphorus (orange) and gold atoms (yellow) are highlighted in space-fill representation to facilitate the visualization of the coordination motif.

Moreover, when the clustering effect of **2** was studied for systems containing different metallic cations (Na^+^, Fe^3+^, or Au^3+^), a clear correlation between cation identity and clustering capability was observed (Figure 4g). Although the monovalent and noncoordinating Na^+^ did not alter at all the clustering capability of the compound (the same number of clustering with the average same size is obtained for both simulations), the presence of trivalent and coordinating Fe^3+^ and Au^3+^ ions strongly altered the self-assembly process, promoting the formation of significantly larger clusters (ca. 70 Å compared to 35 to 45 Å for Na^+^ or metal-free systems). Notably, cluster growth in the gold-containing system occurred earlier in the simulation time frame, beginning at approximately 40 ns, whereas clustering in the Fe^3+^ system emerged later, around 50 ns. This difference suggests a particularly favorable interaction between compound **2** and Au^3+^ cations, consistent with the well-established affinity between phosphine donor groups and gold centers.^34^ Importantly, the resulting metal-associated clusters displayed clear structural differences compared to those formed in the absence of coordinating cations. The supramolecular packing of the gold-containing clusters appeared more compact, with phosphine groups preferentially oriented toward the interior of the assembly, where the metal species were located (Figure 5). In contrast, in metal-free clusters, phosphine groups were distributed in a more isotropical and less orientated fashion, allowing for dispersive interaction arising from the aryl groups to be developed easily and promoting greater exposure to the surrounding solvent. Analysis of the coordination environment further revealed that, well before the end of the simulation time frame, each gold center was surrounded by multiple phosphine groups in a defined spatial arrangement, consistent with strong and persistent coordination interactions typical of phosphine–gold systems.^35^ These observations support the role of gold as an effective structural organizer capable of stabilizing compact supramolecular aggregates through multivalent coordination.

Further comparison of the different simulated systems revealed marked differences in the extent of direct metal–cluster association. Sodium ions exhibited only transient and superficial electrostatic interactions with the supramolecular aggregates, frequently exchanging between clusters and the bulk solvent. In contrast, both Fe^3+^ and Au^3+^ cations occupied well-defined positions within the clusters and remained associated throughout the simulation. In these cases, compound **2** effectively surrounded the metal centers, with phosphine lone pairs oriented toward the metal, generating coordination environments resembling localized binding sites within the supramolecular framework. These results strongly support coordination-driven reorganization as a key factor governing cluster formation and stability in the presence of strongly interacting metal species, particularly for Au^3+^ cations.

## 3 Conclusions

In this work, three phosphine-functionalized squaramides were designed, synthesized, and evaluated as low-molecular-weight supramolecular organogelators. Among them, only compound **2** exhibited robust gelation ability, forming stable, self-supported organogels and alcogels across a broad range of solvents. This behavior highlights the critical role of the molecular structure and the balance between hydrogen bonding and solvophobic interactions in enabling the formation of extended fibrillar networks capable of immobilizing the solvent.

The organogels formed by compound **2** displayed a highly selective response to metal ions. In particular, the addition of Au^3+^ induced rapid and complete gel collapse even at low concentrations, whereas other metal ions produced little or no macroscopic effect or required significantly higher loadings to perturb the gel network. This exceptional responsiveness toward gold is attributed to the strong affinity between this metal and the phosphine functional groups of the gelators. Under the experimental conditions employed, gold derived from Au^3+^ may undergo changes in the coordination environment and nuclearity, potentially involving lower-valent or multinuclear species stabilized by phosphine coordination. Such species are capable of forming strong and multivalent interactions with the gelator, promoting local structural reorganization, densification of supramolecular aggregates, and disruption of the fibrillar network responsible for gel stability.

Molecular dynamics simulations provided molecular-level insight into this process, revealing that gold-containing systems promote the formation of larger and more compact supramolecular clusters stabilized by persistent phosphine–metal coordination. These interactions induce a distinct reorganization of the supramolecular packing, characterized by preferential inward orientation of phosphine groups toward the metal centers. This coordination-driven rearrangement contrasts with the more open, hydrogen bond-dominated assemblies observed in the absence of strongly coordinating metal species and provides a plausible structural basis for the experimentally observed gel destabilization.

Overall, this work demonstrates that the incorporation of phosphine ligands into squaramide-based gelators represents an effective strategy for engineering metal-responsive supramolecular materials. The ability of Au^3+^ cations to selectively disrupt the gel network highlights the potential of these systems as adaptive soft materials capable of transducing specific metal–ligand interactions into macroscopic responses. These findings expand the functional scope of squaramide-based assemblies and establish a foundation for the development of responsive supramolecular materials for metal sensing, controlled disassembly, and stimuli-responsive soft matter applications.

## 4 Experimental Section

### 4.1 Reagents and Materials

Potassium tetrachloroaurate­(III) (KAuCl_4_, 98%), silver nitrate (AgNO_3_, 99%), sodium chloride (NaCl, 99%), copper­(II) chloride dihydrate (CuCl_2_, 99%), calcium chloride (CaCl_2_, 96%), palladium­(II) chloride (PdCl_2_, 99%), europium­(III) chloride (EuCl_3_, 99%), ytterbium­(III) chloride (YbCl_3_, 99%), and chromium­(III) chloride hexahydrate (CrCl_3_, 96%) were purchased from Sigma-Aldrich. All gels were prepared with methanol (≥99.6%, Laboratory Reagent grade from Sigma-Aldrich).

3,4-Dimethoxy-3-cyclobutene-1,2-dione (98%), 3,5-bis­(trifluoromethyl)­aniline (98%), 2-(diphenylphosphino)­ethylamine (95%), 4-(trifluoromethyl)­aniline (98%), 3,5-bis­(trifluoromethyl)­benzylamine (97%), and 2-methoxyethylamine (98%) were purchased from BLD Pharmatech and were used without further purification.

1-Pentamine (98%) and 2-aminoethanol (99%) were purchased from TCI Europe and were used without further purification.

#### 4.1.1 Safety Considerations

No unexpected or unusually hazardous experimental procedures were encountered during this work. All experiments were conducted in accordance with standard laboratory safety practices, using appropriate personal protective equipment and handling all chemicals, including phosphine-containing compounds and metal salts, in a well-ventilated fume hood.

### 4.2 Instrumentation

#### 4.2.1 NMR Spectroscopy

^1^H, ^13^C­{^1^H}-APT, ^19^F, and ^31^P­{^1^H} NMR, including 2D experiments like COSY (^1^H–^1^H), HSQC (^1^H–^13^C), and HMBC (^1^H–^13^C), were recorded at room temperature on a Bruker Avance 400 spectrometer (400.0 MHz for ^1^H, 100.6 MHz for ^13^C­{^1^H}-APT, 376.5 MHz for ^19^F, and 162.0 MHz for ^31^P­{^1^H}), with chemical shifts (δ, ppm) reported relative to the solvent peaks of the deuterated solvent. All J values are given in Hz.

#### 4.2.2 Mass Spectrometry

Mass spectra were recorded on a Bruker Esquire 3000 PLUS with the electrospray (ESI) technique and time-of-flight (Q-TOF) as the analyzer and on a Bruker Microflex (MALDI-TOF).

#### 4.2.3 Mechanical Evaluation of the Hydrogels

**Gel Kinetics.** We investigated the gel kinetics by means of rheological measurements, using a Haake MARS III controlled-stress rheometer (Thermo Fisher Scientific, Waltham, MA, USA). The setup employed a 35 mm diameter parallel-plate sensor. To prevent wall slip, the upper (rotating) plate featured a serrated surface (sensor P35 Ti L S, Thermo Fisher Scientific). The lower plate was specifically designed with a disk-like recessed cavity (or “well”) to facilitate the deposition and containment of the liquid pregel sample. Following the preparation protocol described above, the mixture was poured directly into this cavity. The gelling sample was then subjected to an oscillatory shear strain at a fixed frequency of 1 Hz and a strain amplitude of γ_0_ = 0.001. The evolution of the viscoelastic moduli was then monitored over time at a constant temperature of 25.0 ± 0.1 °C. The chosen strain amplitude was sufficiently low to ensure the formation of the gel microstructure remained unperturbed during measurement.
**Characterization of the Mechanical Properties of the Hydrogels.** The mechanical properties of the fully formed hydrogels were characterized using the same Haake MARS III rheometer and the 35 mm plate–plate geometry described in section (a). All hydrogels were formed within the lower plate cavity of the measuring geometry prior to starting the mechanical characterization to ensure perfect contact and structural integrity. Characterization was carried out at a constant temperature of 25.0 ± 0.1 °C. First, we subjected the hydrogels to amplitude sweeps, for which the frequency of oscillation was kept at 1 Hz and the amplitude of the oscillatory strain, γ_0_, was increased stepwise from 0.0001 to 2. From these measurements, we obtained the values of the storage (*G*′) and loss (*G*″) moduli as a function of γ_0_. Afterward, we performed frequency sweep tests, for which the amplitude of the shear strain was fixed at γ_0_ = 0.0001, and the frequency of oscillation was increased stepwise from 0.1 to 16 Hz. From these measurements, we obtained the values of *G*′ and *G*″ as a function of the frequency (i.e., the mechanical spectra) of the gel. A fresh sample was used for each test (amplitude and frequency sweeps) and experimental conditions, and measurements for at least three different aliquots of the same experimental condition were conducted. Statistical comparison of the mean log10 (*G*′) and log10 (*G″*) values obtained from independent gel replicates was performed using Welch’s *t*-test. No statistically significant differences were detected between both organogels (*p* = 0.12). In this work, we provide the corresponding mean values and standard errors of the measurements.

**Fourier transform infrared (FTIR) spectra** of hydrogels and dried gels were recorded on a Tensor 27 (Bruker, Karlsruhe, Germany) spectrometer in an ATR configuration. The sample was deposited and pressed on a diamond window. Air was collected as the background. The infrared spectra were recorded by accumulating 25 scans, covering the range from 400 cm^–1^ to 4000 cm^–1^ at a resolution of 3 cm^–1^.

#### 4.2.4 Transmission Electron Microscopy (TEM)

Images were recorded with a LIBRA 120 PLUS instrument (Carl Zeiss SMT, Centre for Scientific Instrumentation of the University of Granada, CIC-UGR), operating at 120 kV. A small portion of the synthesized gel was placed on 300-mesh copper grids and incubated for several minutes. Then, the grids were washed with ultrapure water six times (drops of 30 μL) and left to air-dry under ambient conditions for 1 h. TEM samples were not stained (neither with osmium nor with uranyl acetate).

### 4.3 Characterization of Hybrid Materials

#### 4.3.1 Gel Formation

The ability of our compounds to form a gel was explored with several different solvents using the heating–cooling protocol. The heating–cooling technique involves solubilizing the compound by heating it and then allowing it to cool. However, a slight modification was made for our compounds because it was experimentally verified that, after heating the sample, a lower concentration was achieved in almost all solvents if the sample was first placed in an ultrasonic bath and then allowed to cool to room temperature. Gel formation was visually evaluated by an inverted vial method.

All the experiments were carried out in a 2 mL vial. 2.5 mg of squaramide was weighed in a 2 mL vial. Then, 0.5 mL of methanol was added and mixed vigorously by vortexing, heated to 64 °C, and subsequently placed in an ultrasonic bath for 30 s. Finally, the vial was allowed to cool to room temperature.

#### 4.3.2 Metal-Ion Gel Interaction

All solutions were prepared in a 2 mL Eppendorf tube. The inorganic salt was weighed into a 2 mL Eppendorf tube and dissolved in 0.5 mL of distilled water to obtain a stock solution with a concentration of [0.1 M]. This solution was diluted into four different solutions, 0.22 mM (0.025 equiv of metal salt relative to compound **2**), 0.45 mM (0.05 equiv of metal salt relative to compound **2**), 0.67 mM (0.075 equiv of metal salt relative to compound **2**), and 0.89 mM (0.1 equiv of metal salt relative to compound **2**), of the inorganic salt in methanol. Next, 0.1 mL of each of these diluted solutions (separately) was added to four different vials where the gel had previously been formed (2 mL vial, 2.5 mg of compound **2** in 0.5 mL of methanol). This allowed us to study the effect of the metal salt concentration on gel stability. The gels did not break when AgNO_3_, NaCl, CuCl_2_, CaCl_2_, PdCl_2_, EuCl_3_, and YbCl_3_ salts were added, even over a period of months. The gel was immediately destroyed when KAuCl_4_ was added.

#### 4.3.3 Gel-Mixed Compound **2** and Cysteine Formation

All experiments were carried out in a 2 mL vial. Squaramide (2.5 mg, 5.34 μmol) was weighed into the vial. Then, 107 μL of a 0.05 M cysteine solution (5.34 μmol) was added, followed by 393 μL of methanol to reach a total volume of 0.5 mL. The mixture was vigorously mixed using a vortex, heated to 64 °C until complete dissolution, and subsequently placed in an ultrasonic bath for 30 s. Finally, the vial was allowed to cool to room temperature. Gel recovery was evaluated by the inverted vial method. Next, 0.1 mL of 0.89 mM (0.1 equiv of potassium tetrachloroaurate­(III) metal salt relative to compound **2**) was added to the vial where the gel had previously been formed.

#### 4.3.4 Reversibility Study of Gold-Induced Gel Disruption via Cysteine Addition

To investigate the reversibility of the gold-induced gel collapse, a recovery experiment was designed using cysteine as a competitive ligand. The gel was first formed under standard conditions (2.5 mg of compound **2** in 0.5 mL of methanol). Subsequently, KAuCl_4_ (0.1 equiv) was added, leading to gel collapse. After complete disruption, cysteine was introduced into the system as a competing ligand in excess (2.3 equiv relative to Au^3+^) by adding 10 μL of a 0.125 M cysteine solution (prepared by dilution of a 0.25 M stock solution with methanol). The mixture was gently agitated and subjected to ultrasonic treatment for 30 s and then allowed to stand at room temperature. Gel recovery was evaluated by the inverted vial method.

### 4.4 Molecular Dynamics Simulation

The three-dimensional structures of all the studied molecules and methanol were created using Avogadro software,^36^ and the topology file was generated by the online server LigParGen^37^ with the OPLS/CM1A force field.^38,39^ For the metallic cations (Fe^3+^ and Au^3+^), the topology was coined by hand, but the OPLS/CM1A standard ion values were employed for its description.

The studied systems were composed of 51 ligand molecules in a 20 × 20 × 20 nm cubic box, which represents the same concentration employed for the gelification and coordination experiments (5 mg/mL, 9.36 mM for the ligands). The ion concentration was adjusted to represent a proportion of 1:3 ligand/cation for the metals. The methanol solvent was added to each box using the “solvate” command in GROMACS. To estimate the volume of each methanol molecule, the “Calculate Collision Diameter” tool implemented in Chemcraft software^40^ was employed.

MD simulations were carried out using GROMACS 2023 software with the OPLS/CM1A force field. To compare the results and check for any potential force field bias, the CHARMM27 force field was also employed under the same conditions, but these simulations do not give place to any appreciable self-assembly, even when extending them over 50 ns.^41^

Energy minimization was performed using the steepest descent algorithm. To guarantee a strictly minimized system, the cutoff value for the maximum tolerable force was kept under 1000 kJ/mol, and the number of steps was set to a maximum of 150,000. After the minimization, the simulation boxes were equilibrated at both constant volume and pressure. The LINCS algorithm was employed to constrain only the bond lengths involving hydrogen. To achieve the desired temperature of 298 K and a pressure of 1 bar, the modified Berendsen thermostat and the Berendsen barostat algorithm were utilized for 200 ps each. The compressibility was adjusted to represent a diluted water solution (4.50 × 10^–5^) with a Tau parameter of 2 to ensure slow and smooth pressure coupling. To account for long-range interactions, the Particle Mesh Ewald model was used, with a 1 nm cutoff for short-range electrostatic and van der Waals interactions.

After the equilibration phases, all of the simulations were carried out for at least 100 ns.

Clustering analysis was performed using an in-house Python script based on the MD Analysis library and complemented with NumPy for calculating averages. The analysis has been carried out on the ligand-only trajectory file. All these files have been previously treated with the trjconv module to make the ligand molecules whole. This allowed us to guarantee that no misinterpretation of the molecule position will happen, facilitating the search for clusters in a tidier manner. The average cluster size was computed using a 10 Å cutoff distance and averaged over 100 frames for each value to increase the statistical significance.

### 4.5 Synthesis

#### 4.5.1 Synthesis of Compound **1**

To a solution of 3,4-dimethoxy-3-cyclobutene-1,2-dione (29 mg, 0.2 mmol) in methanol (5 mL) was added 3,5-bis­(trifluoromethyl)­aniline (32 μL, 0.2 mmol), and the solution was stirred at room temperature. 80 h later, 2-(diphenylphosphino)­ethylamine was added (46 μL, 0.2 mmol) and the solution was stirred for 24 h at the same temperature. The solution was concentrated under reduced pressure to approximately 1 mL, and Et_2_O (10 mL) was added to precipitate a white solid. The solid was collected by filtration and dried under vacuum to afford product **1** (84 mg, 77% yield).
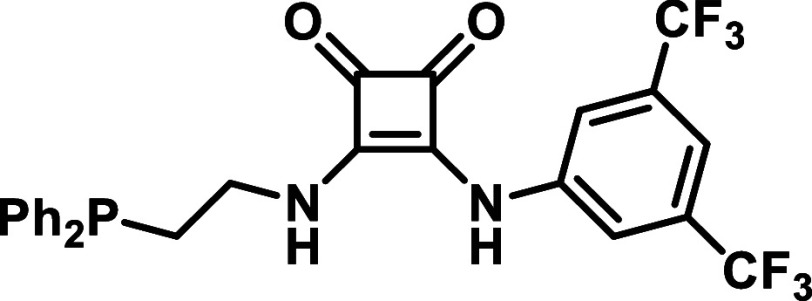


^**1**^ **H NMR (ppm) (400 MHz, DMSO-** ***d***_**6**_): δ = 10.04 (s, 1H, *NH*–Ph); 7.96 (s, 2H, *H*_*ortho*_Ph–CF_3_); 7.79 (s, 1H, *NH*–CH_2_); 7.60 (s, 1H, *H*_*para*_Ph–CF_3_); 7.41 (m, 4H, *H*_*ortho*_PPh_2_); 7.31 (m, 6H, *H*_*meta*_ *+H*_*para*_PPh_2_); 3.72 (br s, 2H, PPh_2_–CH_2_–*CH*_2_); 2.47 (br s, 2H, PPh_2_–*CH*_2_–CH_2_). ^**19**^ **F­{** ^**1**^ **H} NMR (ppm) (376 MHz, DMSO-** ***d***_**6**_): δ = −61.8 (s, 3F, *CF*_3_). ^**31**^ **P­{** ^**1**^ **H} NMR (ppm) (162 MHz, DMSO-** ***d***_**6**_): δ = −22.6 (s, 1P, *PPh*_2_). ^**13**^ **C­{** ^**1**^ **H}-APT (ppm) (100 MHz, DMSO-** ***d***_**6**_): δ = 184.7 (s, 1C, *C=O*); 180.5 (s, 1C, *C=O*); 169.6 (s, 1C, Ph–C=*C–NH*–*CH*_2_); 162.4 (s, 1C, *Ph–NH–C*=C–CH_2_); 141.1 (s, 1C, *C*_*ipso*_–Ph–CF_3_); 137.6 (d, 2C, *C*_*ipso*_ *–*PPh_2_, ^1^ *J*_*CP*_ = 12.8 Hz); 132.5 (d, 4C, *C*_*ortho*_PPh_2_, ^2^ *J*_*CP*_ = 19.0 Hz); 131.2 (q, 1C, *C*_*ipso*_ *–*Ph–CF_3_, ^2^ *J*_*CP*_ = 32.2 Hz); 128.8 (s, 2C, *C*_*para*_PPh_2_); 128.6 (d, 4C, *C*_*meta*_PPh_2_, ^2^ *J*_*CP*_ = 6.8 Hz); 121.8 (q, 2C, *CF*_3_, ^1^ *J*_*CF*_ = 272.9 Hz); 119.1 (s, 2C, *C*_*ortho*_Ph–CF_3_); 114.6 (s, 1C, *C*_*para*_Ph–CF_3_); 41.5 (d, 1C, PPh_2_ *–*CH_2_–*CH*_2_, ^2^ *J*_*CP*_ = 22.6 Hz); 29.3 (d, 1C, PPh_2_–*CH*_2_ *–*CH_2_, ^2^ *J*_*CP*_ = 13.1 Hz). **HRMS (ESI+ μ-TOF): *m*/** ***z*** **(%)=** [M + Na]^+^ calcd for C_26_H_19_F_6_N_2_NaO_2_P: 559.0981; found: 559.0960. mp 232–235 °C.

#### 4.5.2 Synthesis of Compound **2**

To a solution of 3,4-dimethoxy-3-cyclobutene-1,2-dione (29 mg, 0.2 mmol) in methanol (5 mL) was added 4-(trifluoromethyl)­aniline (25 μL, 0.2 mmol), and the solution was stirred at room temperature. 64 h later, 2-(diphenylphosphino)­ethylamine was added (46 μL, 0.2 mmol) and the solution was stirred for 24 h at the same temperature. The solution was concentrated under reduced pressure to approximately 1 mL, and Et_2_O (10 mL) was added to precipitate a white solid. The solid was collected by filtration and dried under vacuum to afford product **2** (94 mg, 98% yield).
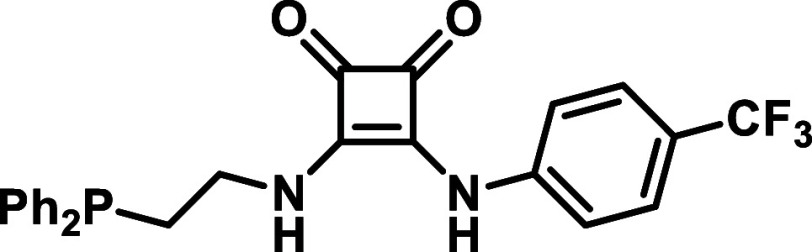


^**1**^ **H NMR (ppm) (400 MHz, DMSO-** ***d***_**6**_): δ = 9.88 (s, 1H, *NH*–Ph); 7.85 (s, 1H, *NH*–CH_2_); 7.66 (s, 2H, *H*_*ortho*_Ph–CF_3_); 7.58 (s, 2H, *H*_*meta*_Ph–CF_3_); 7.45 (m, 4H, *H*_*ortho*_PPh_2_); 7.34 (m, 6H, *H*_*meta*_ *+H*_*para*_PPh_2_); 3.74 (m, 2H, PPh_2_ *–*CH_2_–*CH*_2_); 2.50 (m, 2H, PPh_2_ *–CH*_2_ *–*CH_2_). ^**19**^ **F­{** ^**1**^ **H} NMR (ppm) (376 MHz, DMSO-** ***d***_**6**_): δ = −60.2 (s, 3F, *CF*_3_). ^**31**^ **P­{** ^**1**^ **H} NMR (ppm) (162 MHz, DMSO-** *d*_**6**_): δ = −22.4 (s, 1P, *PPh*_2_). ^**13**^ **C­{** ^**1**^ **H}-APT (ppm) (100 MHz, DMSO-** ***d***_**6**_): δ = 184.6 (s, 1C, *C = O*); 180.2 (s, 1C, *C=O*); 169.4 (s, 1C, Ph–C=*C–NH*–*CH*_2_); 162.9 (s, 1C, *Ph–NH–C*=C–CH_2_); 142.6 (s, 1C, *C*_*ipso*_–Ph–CF_3_); 137.5 (d, 2C, *C*_*ipso*_–PPh_2_, ^1^ *J*_*CP*_ = 12.8 Hz); 132.3 (d, 4C, *C*_*ortho*_PPh_2_, ^2^ *J*_*CP*_ = 19.0 Hz); 128.8 (s, 2C, *C*_*para*_PPh_2_); 128.6 (d, 4C, *C*_*meta*_PPh_2_, ^2^ *J*_*CP*_ = 6.8 Hz); 126.6 (s, 2C, *C*_*ortho*_Ph–CF_3_); 123.1 (m, 2C, *CF*_3_); 121.8 (q, 1C, *C*_*ipso*_–Ph–CF_3_, ^2^ *J*_*CP*_ = 32.0 Hz); 117.9 (s, 2C, *C*_*meta*_Ph–CF_3_); 41.5 (d, 1C, PPh_2_–CH_2_–*CH*_2_, ^2^ *J*_*CP*_ = 22.6 Hz); 29.2 (d, 1C, PPh_2_–*CH*_2_–CH_2_, ^2^ *J*_*CP*_ = 13.2 Hz). **HRMS (ESI+ μ-TOF): *m*/** ***z*** **(%)=** [M + Na]^+^ calcd for C_25_H_20_F_3_N_2_NaO_2_P: 491.1107; found: 491.1090. mp 202–205 °C.

#### 4.5.3 Synthesis of Compound **3**

To a solution of 3,4-dimethoxy-3-cyclobutene-1,2-dione (29 mg, 0.2 mmol) in methanol (5 mL) was added 3,5-bis­(trifluoromethyl)­benzylamine (50 mg, 0.2 mmol), and the solution was stirred at room temperature. 21 h later, 2-(diphenylphosphino)­ethylamine was added (46 μL, 0.2 mmol) and the solution was stirred for 24 h at the same temperature. The solution was concentrated under reduced pressure to approximately 1 mL, and Et_2_O (10 mL) was added to precipitate a white solid. The solid was collected by filtration and dried under vacuum to afford product **3** (107 mg, 95% yield).
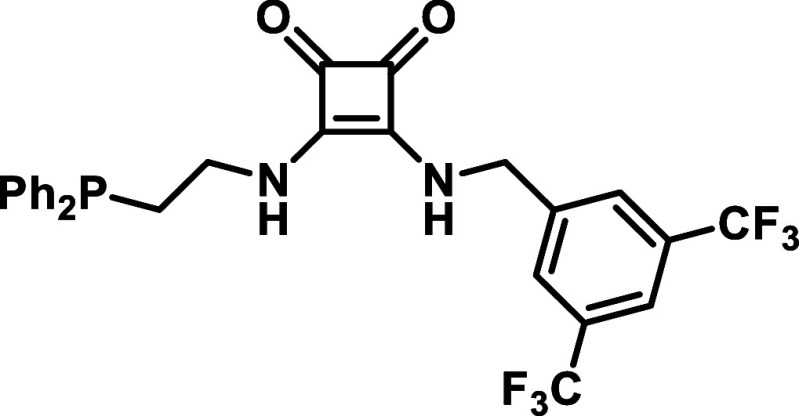


^**1**^ **H NMR (ppm) (400 MHz, DMSO-** ***d***_**6**_): δ = 8.06 (m, 3H, *H*_*ortho*_Ph–CF_3_+*H*_*para*_Ph–CF_3_); 7.33 (m, 10H, *PPh*_2_); 4.86 (s, 2H, NH–*CH*_2_–Ph–CF_3_); 3.63 (t, 2H, PPh_2_–CH_2_–*CH*_2_, ^3^ *J*_*H–H*_ = 7.3 Hz); 2.42 (s, 2H, PPh_2_–*CH*_2_–CH_2_). ^**1**^ **H NMR (ppm) (400 MHz, CD**_**3**_ **CN):** δ = 7.97 (s, 2H, *H*_*ortho*_Ph–CF_3_); 7.46–7.36 (m, 11H, *H*_*para*_Ph–CF_3_ *+PPh*_2_); 6.30 (s, 1H, *NH*); 6.12 (s, 1H, *NH*); 4.86 (d, 2H, NH–*CH*_2_–Ph–CF_3_, ^3^ *J*_*H–H*_ = 6.0 Hz); 3.72 (m, 2H, PPh_2_–CH_2_–*CH*_2_); 2.45 (m, 2H, PPh_2_–*CH*_2_–CH_2_). ^**19**^ **F­{** ^**1**^ **H} NMR (ppm) (376 MHz, DMSO-** ***d***_**6**_): δ = −61.3 (s, 6F, *CF*_3_). ^**31**^ **P­{** ^**1**^ **H} NMR (ppm) (162 MHz, DMSO-** ***d***_**6**_): δ = −22.4 (br s, 1P, *PPh*_2_). ^**13**^ **C­{** ^**1**^ **H}-APT (ppm) (100 MHz, DMSO-** ***d***_**6**_): δ = 182.8 (s, 1C, *C=O*); 182.5 (s, 1C, *C=O*); 167.9 (s, 1C, Ph–C=*C*–*CH*_2_); 167.2 (s, 1C, *Ph–C*=C–CH_2_); 142.6 (s, 1C, *C*_*ipso*_ *–Ph*); 137.6 (d, 2C, *C*_*ipso*_PPh_2_, ^1^ *J*_*CP*_ = 12.9 Hz); 132.5 (d, 4C, *C*_*ortho*_PPh_2_, ^2^ *J*_*CP*_ = 18.9 Hz); 130.3 (q, 2C, *C*_*ipso*_ *C–CF*_3_, ^2^ *J*_*CF*_ = 32.7 Hz); 128.7 (s, 1C, *C*_*para*_PPh_2_); 128.6 (s br, 4C, *C*_*meta*_PPh_2_, ^3^ *J*_*CP*_ = 6.7 Hz); 128.4 (s, 2C, *C*_*ortho*_Ph–CF_3_); 124.7 (q, 2C, *CF*_3_, ^1^ *J*_*CF*_ = 272.9 Hz); 121.2 (s, 1C, *C*_*para*_Ph–CF_3_); 45.7 (s, 1C, NH–*CH*_2_–Ph–CF_3_); 40.8 (s, 1C, PPh_2_–CH_2_–*CH*_2_, ^2^ *J*_*CP*_ = 23.1 Hz); 29.4 (s, 1C, PPh_2_–*CH*_2_–CH_2_, ^1^ *J*_*CP*_ = 13.2 Hz). **HRMS (ESI+ μ-TOF): *m*/*z*(%)=** [M + Na]^+^ calcd for C_27_H_21_F_6_N_2_NaO_2_P: 573.1137; found: 573.1115. mp 227–230 °C.

#### 4.5.4 Synthesis of Compound **4**

To a solution of 3,4-dimethoxy-3-cyclobutene-1,2-dione (29 mg, 0.2 mmol) in methanol (5 mL) was added 4-(trifluoromethyl)­aniline (25 μL, 0.2 mmol), and the solution was stirred at room temperature. 21 h later, 2-aminoethanol was added (12 μL, 0.2 mmol) and the solution was stirred for 24 h at the same temperature. The solution was concentrated under reduced pressure to approximately 1 mL, and Et_2_O (10 mL) was added to precipitate a white solid. The solid was collected by filtration and dried under vacuum to afford product **4** (43 mg, 70% yield).
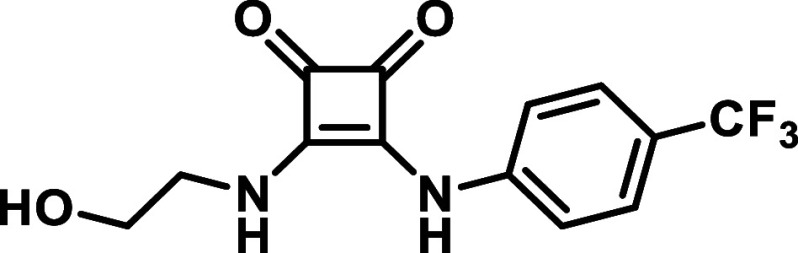


^**1**^ **H NMR (ppm) (400 MHz, DMSO-** ***d***_**6**_): δ = 10.01 (s, 1H, *NH*–Ph); 7.91 (s, 1H, *NH*–CH_2_); 7.67–7.61 (m, 4H, *Ph*); 5.03 (s, 1H, *OH*); 3.67 (m, 2H, OH–CH_2_–*CH*_2_–NH); 3.58 (m, 2H, OH–*CH*_2_–CH_2_–NH). ^**19**^ **F­{** ^**1**^ **H} NMR (ppm) (376 MHz, DMSO-** ***d***_**6**_): δ = −60.1 (s, 3F, *CF*_3_). ^**13**^ **C­{** ^**1**^ **H}-APT (ppm) (100 MHz, DMSO-** ***d***_**6**_): δ = 184.8 (s, 1C, *C=O*); 180.1 (s, 1C, *C=O*); 169.8 (s, 1C, Ph–C=*C*–*CH*_2_); 162.9 (s, 1C, *Ph–C*=C–CH_2_); 142.7 (s, 1C, *C*_*ipso*_ *–Ph*); 125.8 (s, 2C, *C*_*ortho*_Ph–CF_3_); 122.7 (q, 2C, *CF*_3_, ^1^ *J*_*CF*_ = 271.2 Hz); 122.7 (q, 1C, *C*_*ipso*_ *–Ph–CF*_3_, ^2^ *J*_*CP*_ = 32.1 Hz); 117.8 (s, 2C, *C*_*meta*_Ph–CF_3_); 60.5 (s, 2H, OH–*CH*_2_–CH_2_–NH); 46.3 (s, 2H, OH–CH_2_–*CH*_2_–NH). **HRMS (ESI+): *m*/** ***z*** **[M** + H]**^+^** calcd for C_13_H_12_F_3_N_2_O_3_: 301.0795; found: 301.0798. HRMS (ESI+): *m*/*z* [M + Na]^+^ calcd for C_13_H_11_F_3_N_2_NaO_3_: 323.0614; found: 323.0616. mp 278–281 °C.

#### 4.5.5 Synthesis of Compound **5**

To a solution of 3,4-dimethoxy-3-cyclobutene-1,2-dione (29 mg, 0.2 mmol) in methanol (5 mL) was added 4-(trifluoromethyl)­aniline (25 μL, 0.2 mmol), and the solution was stirred at room temperature. 21 h later, 2-methoxyethylamine was added (17 μL, 0.2 mmol) and the solution was stirred for 24 h at the same temperature. The solution was concentrated under reduced pressure to approximately 1 mL, and Et_2_O (10 mL) was added to precipitate a white solid. The solid was collected by filtration and dried under vacuum to afford product **5** (42 mg, 65% yield).
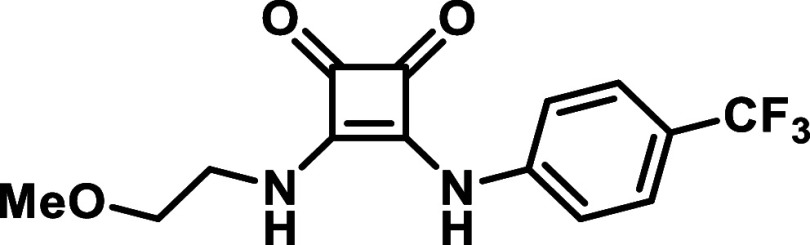


^**1**^ **H NMR (ppm) (400 MHz, DMSO-** ***d***_**6**_): δ = 9.99 (s, 1H, *NH*–Ph); 7.88 (s, 1H, *NH*–CH_2_); 7.67–7.62 (m, 4H, *Ph*); 3.78 (s, 2H, MeO–CH_2_–*CH*_2_–NH); 3.52 (t, 2H, MeO–*CH*_2_–CH_2_–NH, ^3^ *J*_*CP*_ = 5.0 Hz); 3.32 (s, 3H, *OMe*). ^**19**^ **F­{** ^**1**^ **H} NMR (ppm) (376 MHz, DMSO-** ***d***_**6**_): δ = −60.1 (s, 3F, *CF*_3_). ^**13**^ **C­{** ^**1**^ **H}-APT (ppm) (100 MHz, DMSO-** ***d***_**6**_): δ = 184.7 (s, 1C, *C=O*); 180.2 (s, 1C, *C=O*); 169.7 (s, 1C, Ph–C=*C*–*CH*_2_); 162.9 (s, 1C, *Ph–C*=C–CH_2_); 142.7 (s, 1C, *C*_*ipso*_ *Ph*); 126.7 (s, 2C, *C*_*ortho*_Ph–CF_3_); 125.8 (q, 2C, *CF*_3_, ^1^ *J*_*CF*_ = 271.2 Hz); 122.1 (q, 1C, *C*_*ipso*_ *Ph–CF*_3_, ^2^ *J*_*CP*_ = 31.9 Hz); 117.9 (s, 2C, *C*_*meta*_Ph–CF_3_); 71.3 (s, 2H, OMe–*CH*_2_–CH_2_–NH); 58.0 (s, 1C, *OMe*); 43.5 (s, 2H, OMe–CH_2_–*CH*_2_–NH). **HRMS (ESI+): *m*/** ***z*** **[M+H**]^+^ calcd for C_14_H_14_F_3_N_2_O_3_: 315.0951; found: 315.0952. HRMS (ESI+): *m*/*z* [M + Na]^+^ calcd for C_14_H_13_F_3_N_2_NaO_3_: 337.0770; found: 337.0772. mp 276–279 °C.

#### 4.5.6 Synthesis of Compound **6**

To a solution of 3,4-dimethoxy-3-cyclobutene-1,2-dione (29 mg, 0.2 mmol) in methanol (5 mL) was added 4-(trifluoromethyl)­aniline (25 μL, 0.2 mmol), and the solution was stirred at room temperature. 21 h later, 1-pentamine was added (23 μL, 0.2 mmol) and the solution was stirred for 24 h at the same temperature. The solution was concentrated under reduced pressure to approximately 1 mL, and Et_2_O (10 mL) was added to precipitate a white solid. The solid was collected by filtration and dried under vacuum to afford product **6** (56 mg, 84% yield).
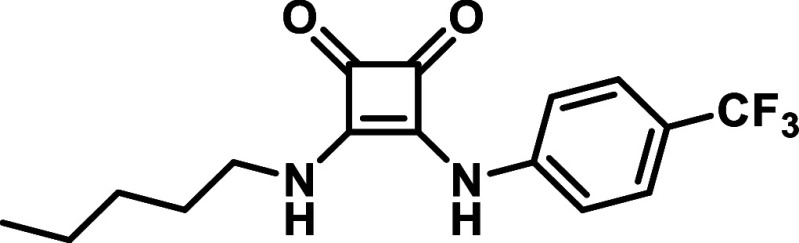


^**1**^ **H NMR (ppm) (400 MHz, DMSO-** ***d***_**6**_): δ = 9.88 (s, 1H, *NH*–Ph); 7.72 (s, 1H, *NH*–CH_2_); 7.66–7.61 (m, 4H, *Ph*); 3.59 (m, 2H, CH_3_–CH_2_–CH_2_–CH_2_–*CH*_2_–NH); 1.57 (m, 2H, CH_3_–CH_2_–CH_2_–*CH*_2_–CH_2_–NH); 1.31 (m, 4H, CH_3_–*CH*_2_ *–CH*_2_–CH_2_–CH_2_–NH); 0.88 (t, 3H, *CH*_3_–CH_2_–CH_2_–CH_2_–CH_2_–NH, ^3^ *J*_*HH*_ = 6.5 Hz). ^**19**^ **F­{** ^**1**^ **H} NMR (ppm) (376 MHz, DMSO-** ***d***_**6**_): δ = −60.1 (s, 3F, *CF*_3_). ^**13**^ **C­{** ^**1**^ **H}-APT (ppm) (100 MHz, DMSO-** ***d***_**6**_): δ = 184.7 (s, 1C, *C*=*O*); 180.0 (s, 1C, *C*=*O*); 169.7 (s, 1C, Ph–C=*C*–*CH*_2_); 162.8 (s, 1C, *Ph–C*=C–CH_2_); 142.8 (s, 1C, *C*_*ipso*_ *–Ph*); 126.6 (s, 2C, *C*_*ortho*_Ph–CF_3_); 125.8 (q, 2C, *CF*_3_, ^1^ *J*_*CF*_ = 271.1 Hz); 122.4 (q, 1C, *C*_*ipso*_ *–Ph–CF*_3_, ^2^ *J*_*CP*_ = 32.1 Hz); 117.9 (s, 2C, *C*_*meta*_Ph–CF_3_); 43.7 (s, 1C, CH_3_–CH_2_–CH_2_–CH_2_–*CH*_2_–NH); 30.2 (s, 1C, CH_3_–CH_2_–CH_2_–*CH*_2_–CH_2_–NH); 28.0, 21.7 (s, 2C, CH_3_–*CH*_2_ *–CH*_2_–CH_2_–CH_2_–NH); 13.8 (s, 1C, *CH*_3_–CH_2_–CH_2_–CH_2_–CH_2_–NH). **HRMS (ESI+): *m*/** ***z*** [M + H]^+^ calcd for C_16_H_18_F_3_N_2_O_2_: 327.1315; found: 327.1323. mp 300–303 °C.

### 4.6 Higher-Scale Squaramide **6**

To a solution of 3,4-dimethoxy-3-cyclobutene-1,2-dione (145 mg, 1 mmol) in methanol (5 mL) was added 4-(trifluoromethyl)­aniline (129 μL, 1 mmol) and the mixture was stirred at room temperature for 48 h. After this period, 1-pentylamine (117 μL, 1 mmol) was added, and the reaction was stirred for an additional 24 h under the same conditions. A precipitate formed, which was collected by filtration, washed with cold MeOH, and dried under vacuum to afford product **6** (250 mg, 75% yield).

## Supplementary Material



## Data Availability

The data underlying this study are available in the published article and its Supporting Information.

## Notes

Tables S1–S4, gel formation test; Figures S1–S3, rheological experiments; Figures S4–S6, IR spectra of compound **2** (solid, gel, and xerogel); Figures S7–S26, TEM images of **2** organogel under different conditions; Figure S27, ^31^P­{^1^H} NMR experiment; and Figures S28–S49, NMR spectra copies (PDF)
