# Supplementary material for: Phosphine-Functionalized Squaramides as Responsive Organogelators: Structure, Gelation, and Metal-Ion Sensing
Source: J Org Chem. 2026 Jul 14;91(29):9959–71. doi: 10.1021/acs.joc.6c00455 (PMC13411053; doi:10.1021/acs.joc.6c00455)
Supplement: Supplementary file 1 [file jo6c00455_si_001.pdf]

SUPPORTING INFORMATION

Phosphine-Functionalized Squaramides as Responsive Organogelators: Structure, Gelation, and Metal Ion Sensing

Daniel Salvador-Gil,[a,b,c,#] Sara Illescas-Lopez,[d,#] Raquel P. Herrera,[e] Federico Movilla,[b,f] David Díaz Díaz,[g,h] Modesto T. López-López,[i,j] M. Concepción Gimeno,[c,*] Araceli G. Campaña,[a,b,*] Luis Álvarez de Cienfuegos.[a,b,j,*]

[a] Universidad de Granada (UGR), Departamento de Química Orgánica, C. U. Fuentenueva, Avda. Severo Ochoa s/n, E-18071 Granada, Spain.

[b] Unidad de Excelencia Química Aplicada a Biomedicina y Medioambiente (UEQ), Universidad de Granada, E-18071, Granada, Spain.

[c] Departamento de Química Inorgánica, Instituto de Síntesis Química y Catálisis Homogénea (ISQCH), CSIC-Universidad de Zaragoza, Pedro Cerbuna 12, 50009 Zaragoza, Spain.

[d] Center for Cooperative Research in Biomaterials (CIC biomaGUNE), Basque Research and Technology Alliance (BRTA), Paseo de Miramón 194, 20014 Donostia-San Sebastián, Spain.

[e] Laboratorio de Organocatálisis Asimétrica. Departamento de Química Orgánica, Instituto de Síntesis Química y Catálisis Homogénea (ISQCH), CSIC-Universidad de Zaragoza, Pedro Cerbuna 12, 50009 Zaragoza, Spain.

[f] Nanoscopy-UGR Laboratory, Faculty of Pharmacy, University of Granada, E-18071 Granada, Spain.

[g] Departamento de Química Orgánica, Universidad de La Laguna, Avda. Astrofísico Francisco Sánchez 3, La Laguna 38206, Tenerife, Spain.

[h] Instituto Universitario de Bio-Orgánica Antonio González, Universidad de La Laguna, Avda. Astrofísico Francisco Sánchez 2, La Laguna 38206, Tenerife, Spain.

[i] Universidad de Granada (UGR), Departamento de Física Aplicada, C. U. Fuentenueva, Avda. Severo Ochoa s/n, E-18071 Granada, Spain.

[j] Instituto de Investigación Biosanitaria ibs.GRANADA Av. De Madrid, 15, 18016, Granada, Spain.

Index

Organogel formation test S3

Rheological experiment S6

ATR-FTIR S7

TEM images S9

^{31}P NMR Experiment S17

NMR spectra for squaramides 1-6 ....S18

Organogel formation test

Table S1. Gelation tests with additional solvents for squaramide **1**.

Entry	Solvent	Phase[a]	Visual appearance	Concentration (mg/mL)	Protocol
1	Acetonitrile	P	Opaque	5	Heating-Ultrasound
2	Toluene	P	Opaque	5	Heating-Ultrasound
3	Chloroform	P	Opaque	5	Heating-Ultrasound
4	Ethanol	G+U	Opaque	10	Heating-Ultrasound
5	Butanol	G+U	Opaque	16.4	Heating-Ultrasound
6	Methanol	G+U	Opaque	10	Heating-Ultrasound
7	2-propanol	P	Opaque	5	Heating-Ultrasound

[a] Abbreviations: G = gel; U = undissolved gelator; P = precipitate. [b] Concentration used for the experiments.

Table S2. Gelation tests with additional solvents for squaramide **3**.

Entry	Solvent	Phase[a]	Visual appearance	Concentration (mg/mL)	Protocol
1	Acetonitrile	G+U	Opaque	5	Heating-Ultrasound
2	Toluene	G+U	Opaque	5	Heating-Ultrasound
3	Methanol	G+U	Opaque	5	Heating-Ultrasound
4	Ethyl acetate	G+U	Opaque	4	Heating-Ultrasound
5	Nitromethane	G+U	Opaque	4	Heating-Ultrasound

[a] Abbreviations: G = gel; U = undissolved gelator. [b] Concentration used for the experiments.

Table S3. Gelation tests with additional solvents for squaramide **2**.

Entry	Solvent	Phase[a]	Visual appearance	SMG[b]	CGC[c,d] (mg/mL)	Protocol	Stability
1	Methanol	G	Opaque	2895	4	Heating-Ultrasound	2 months
2	Acetonitrile	G	Translucent	4990	2	Heating-Ultrasound	3 months
3	Toluene	P	-	-		Heating-Ultrasound	-
4	Chloroform	P	-	-		Heating-Ultrasound	-
5	Butanol	G	Opaque	510	10	Heating-Ultrasound	-
6	Ethanol	G+U	-	-		Heating-Ultrasound	-
7	2-propanol	P	-	-		Heating-Ultrasound	-
8	5-phenyl-1-pentanol	G	Translucent	550	5	Heating-Ultrasound	2 months
9	3-phenyl-2-propyn-1-ol	-	-	-		Heating-Ultrasound	-
10	3-Hydroxypropionitrile	G	Opaque	-		Heating-Ultrasound	3 months
11	1-pentanol	G	Translucent	2895	2	Heating-Ultrasound	4 months
12	2,2,2-Trifluoroethanol	-	-	-		Heating-Ultrasound	2 months
13	Tetrahydrofurfuryl alcohol	-	-	-		Heating-Ultrasound	-
14	5-hexen-1-ol	G	Opaque	155	25	Heating-Ultrasound	2 months
15	Butan-2-ol	G	Translucent	1003	5	Heating-Ultrasound	3 months
16	Benzyl alcohol	-	-	-		Heating-Ultrasound	-
17	*tert*-butyl alcohol	G	Translucent	486	10	Heating-Ultrasound	2 months

[a] Abbreviations: G = gel; U = undissolved gelator; P = precipitate. [b] SMG (solvent molecules per gelator molecule) is calculated from the bulk molar ratio at the CGC. This dimensionless value reflects the global solvent-to-gelator composition under the experimental conditions employed and does not imply specific stoichiometric association, binding, or dynamic immobilization of solvent molecules. Therefore, SMG values should be compared only as

relative compositional indicators under comparable experimental conditions. [c] CGC = Critical gelation concentration (mg/mL). [d] When not indicated, the concentration tested was 5mg/mL in all cases.

Table S4. Gelation tests with additional solvents for squaramides **4**, **5**, and **6**.[a]

Entry	Solvent	**4**	**5**	**6**
1	Toluene	Insoluble	Insoluble	Insoluble
2	Acetonitrile	Aggregate[b]	Insoluble	Aggregate[b]
3	Methanol	Insoluble	Aggregate[b]	Aggregate[b]
4	Chloroform	Insoluble	Insoluble	Insoluble

[a] All experiments were performed at concentrations between 5 and 10 mg/mL, but no gel formation was observed. [b] Visible irregular solid in the solution.

Rheological experiment

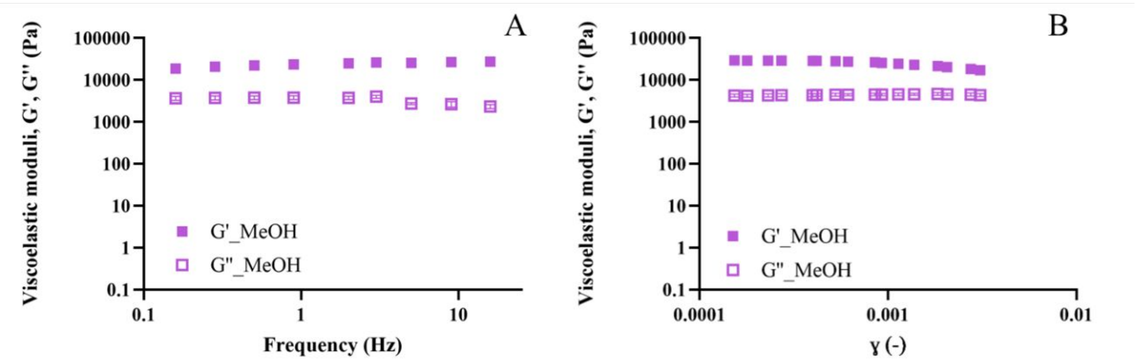

Figure S1. Viscoelastic moduli as a function of the A) frequency and B) oscillatory amplitude for organogel **2** (5 mg/mL) in methanol**.**

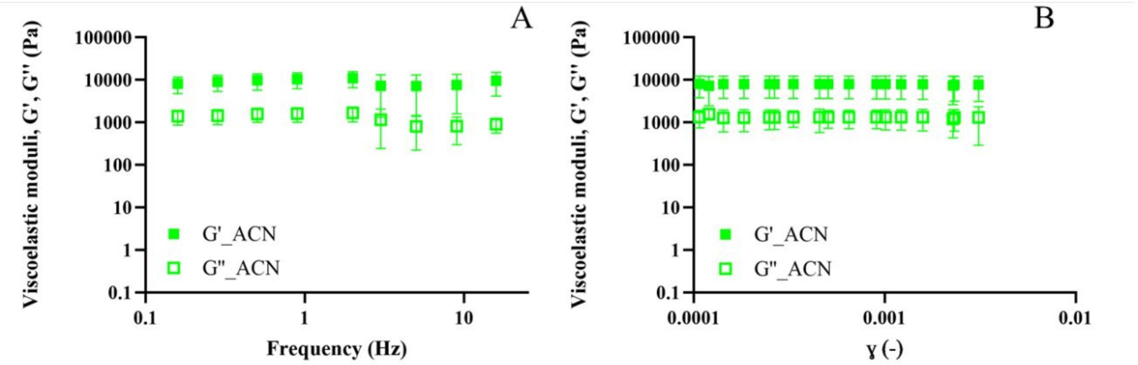

Figure S2. Viscoelastic moduli as a function of the A) frequency and B) oscillatory amplitude for organogel **2** (5 mg/mL) in acetonitrile**.**

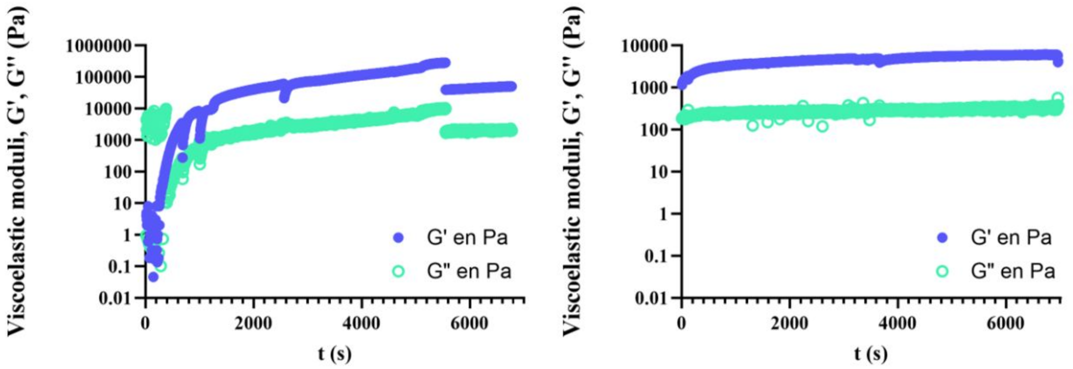

Figure S3. Viscoelastic moduli as a function of time for organogel **2** (5 mg/mL) in methanol**.**

ATR-FTIR

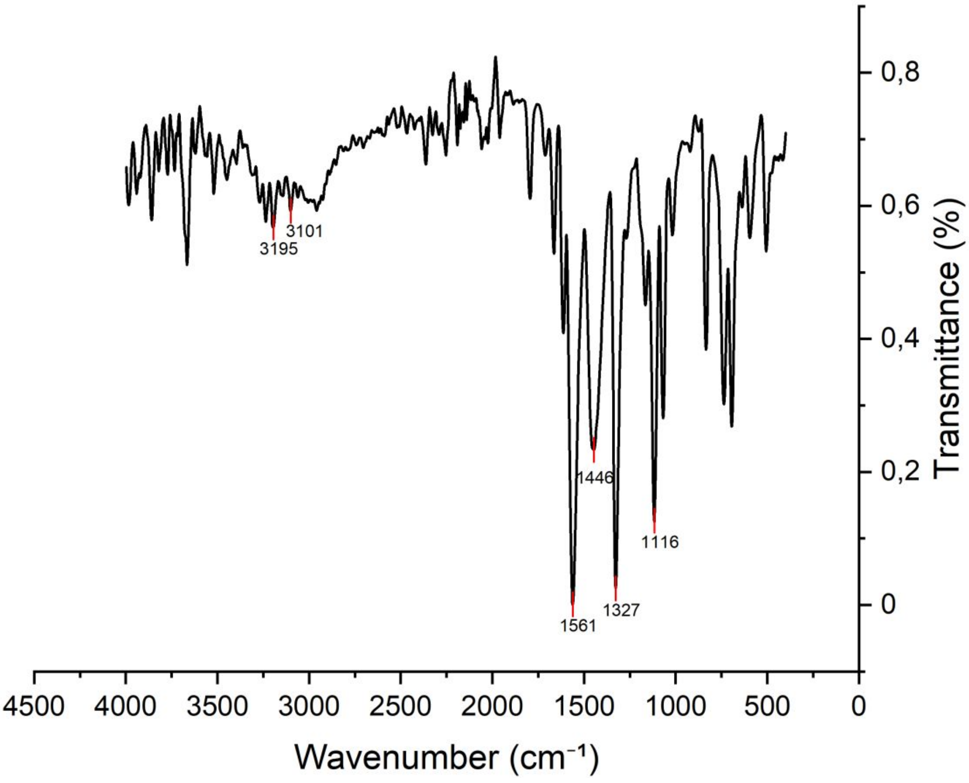

Figure S4. IR spectra of solid compound **2**.

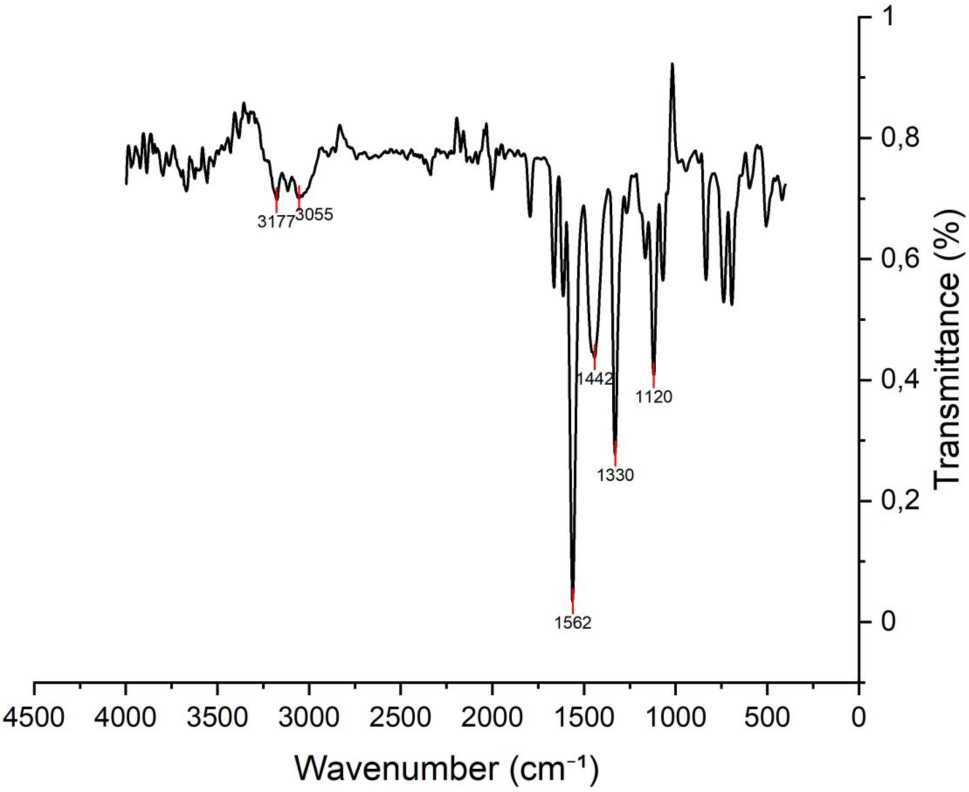

Figure S5. IR spectra of gel **2** (5 mg/mL).

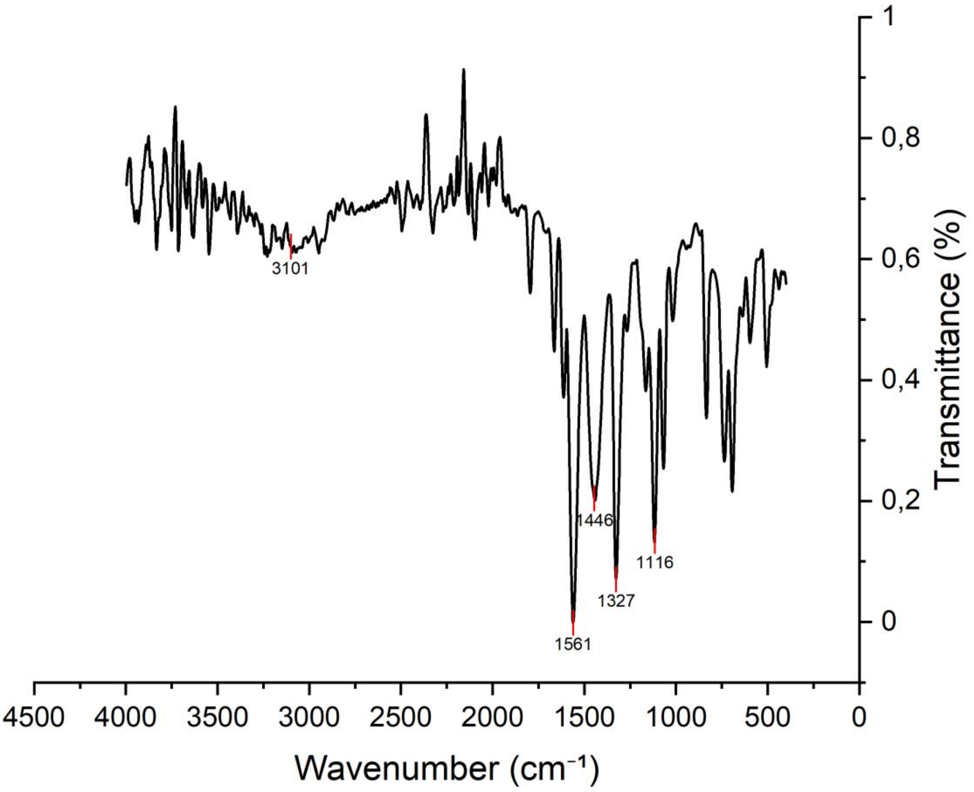

Figure S6. IR spectra of xerogel **2**.

TEM images

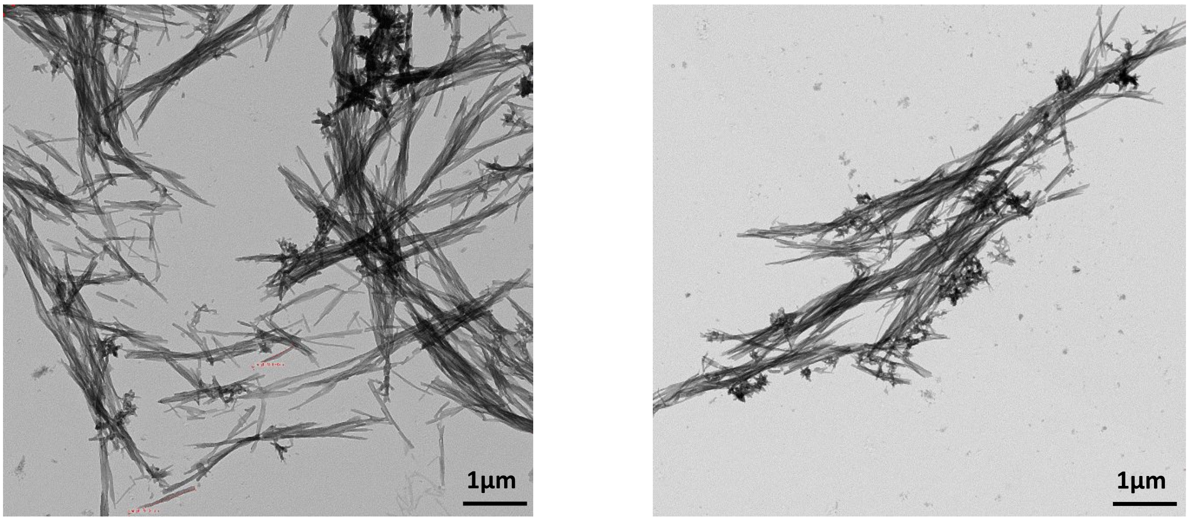

Figure S7. TEM images of organogel **2** (5 mg/mL) in methanol.

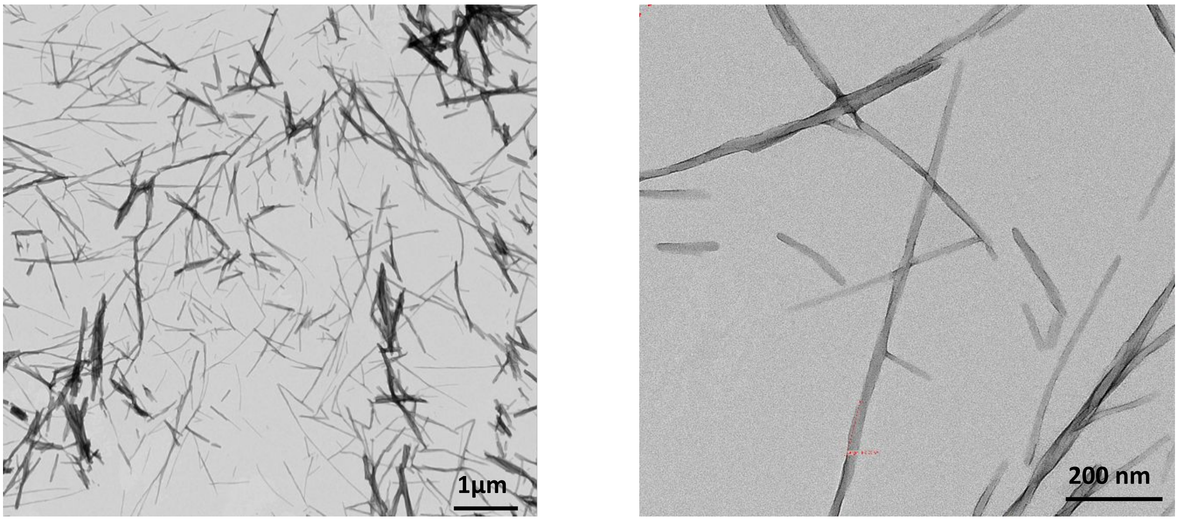

Figure S8. TEM images of organogel **2** (5 mg/mL) after sodium chlorine addition.

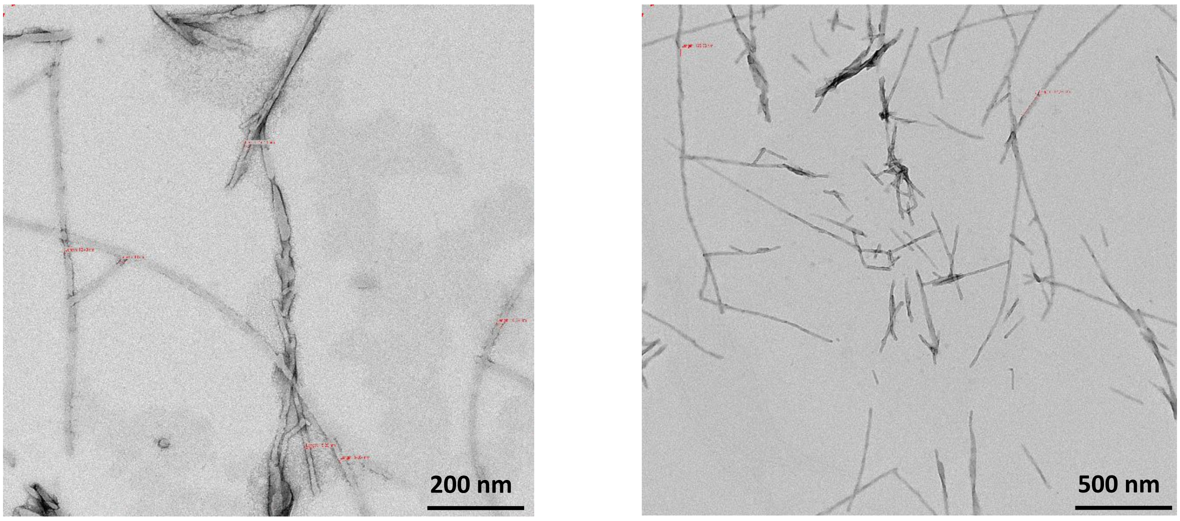

Figure S9. TEM images of organogel **2** (5 mg/mL) after calcium chloride addition.

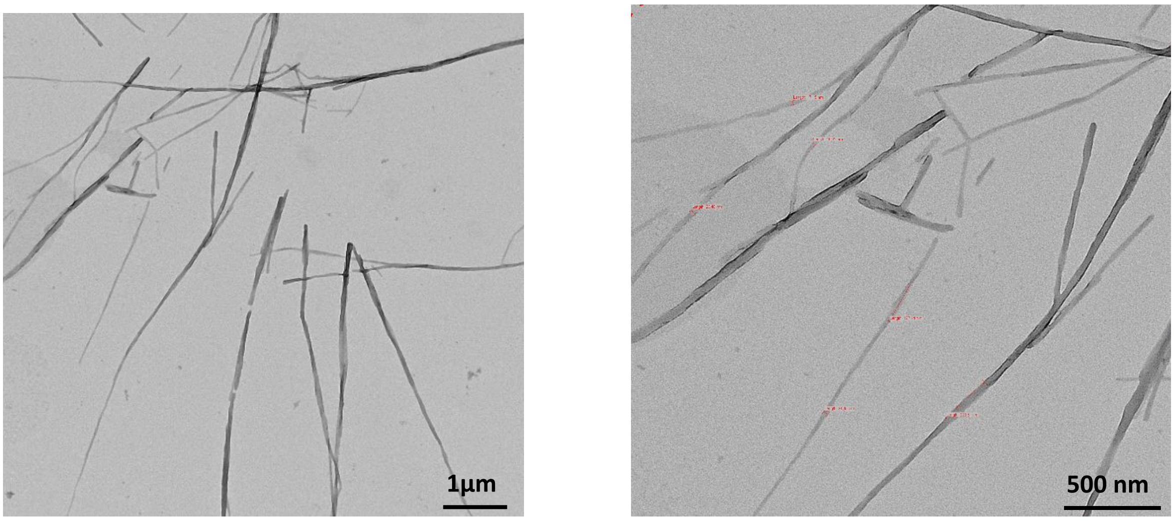

Figure S10. TEM images of organogel **2** (5 mg/mL) after copper(II) chloride dihydrate addition.

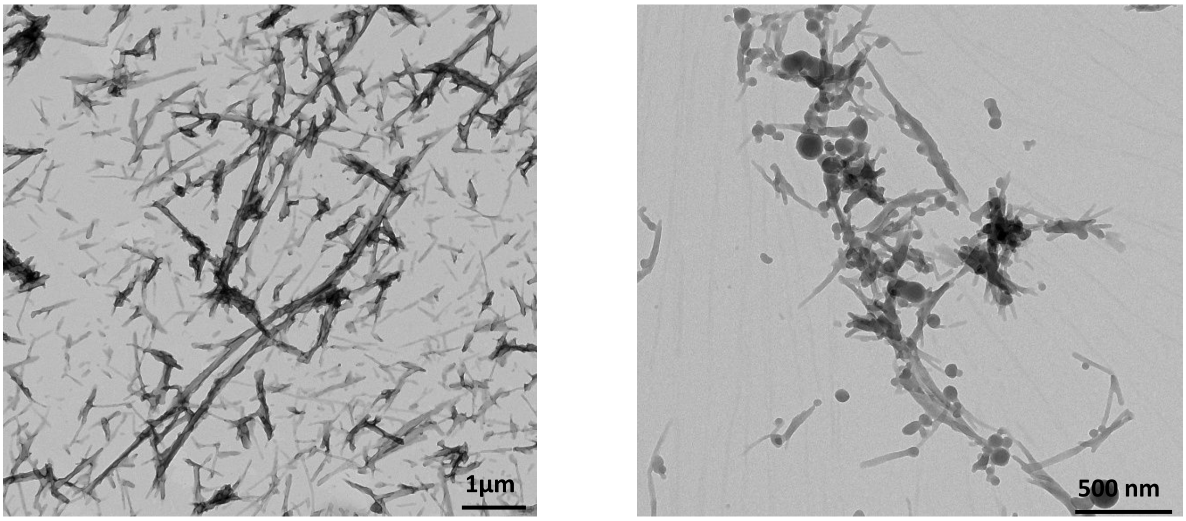

Figure S11. TEM images of organogel **2** (5 mg/mL) after palladium (II) chloride addition.

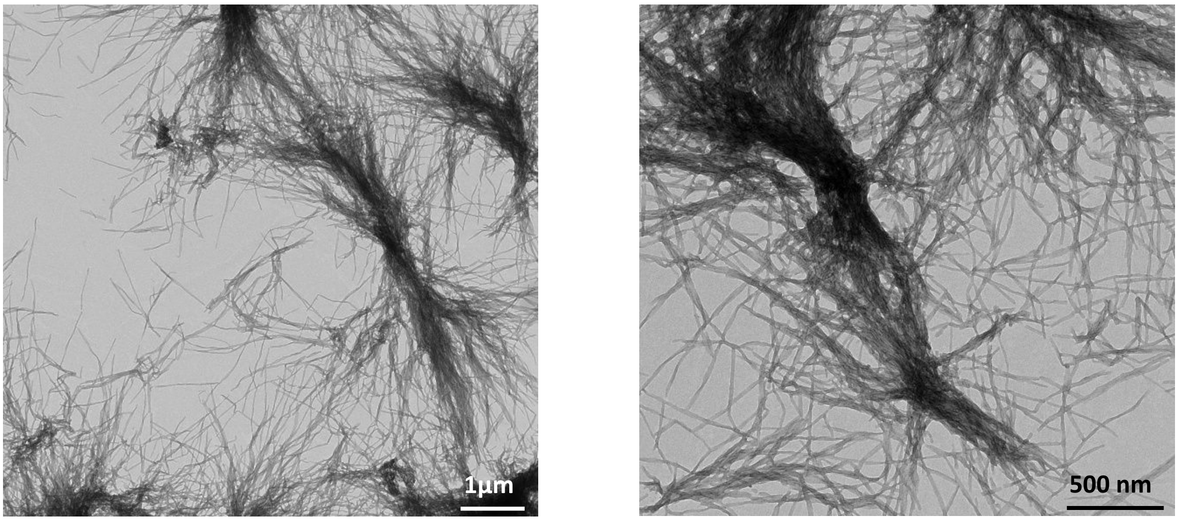

Figure S12. TEM images of organogel **2** (5 mg/mL) after silver nitrate addition.

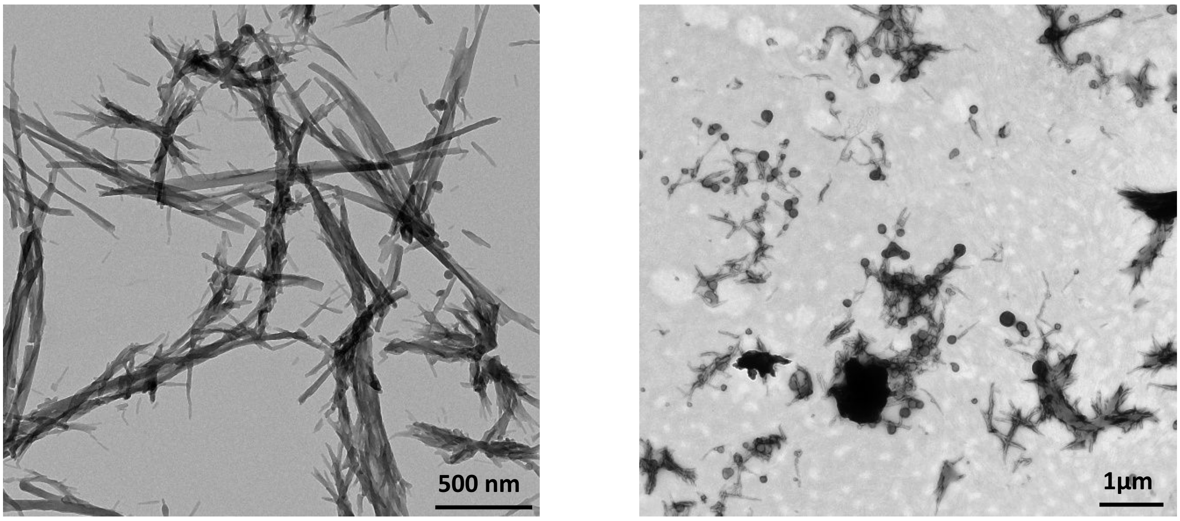

Figure S13. TEM images of organogel **2** (5 mg/mL) after potassium tetrachloroaurate(III), addition.

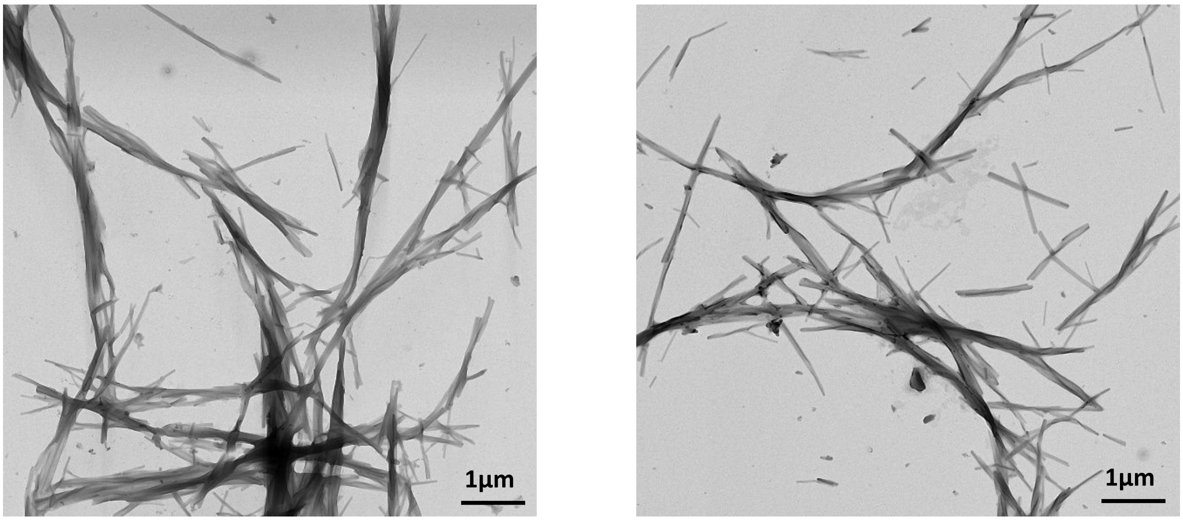

Figure S14. TEM images of organogel **2** (5 mg/mL) after chromium(III) chloride hexahydrate addition.

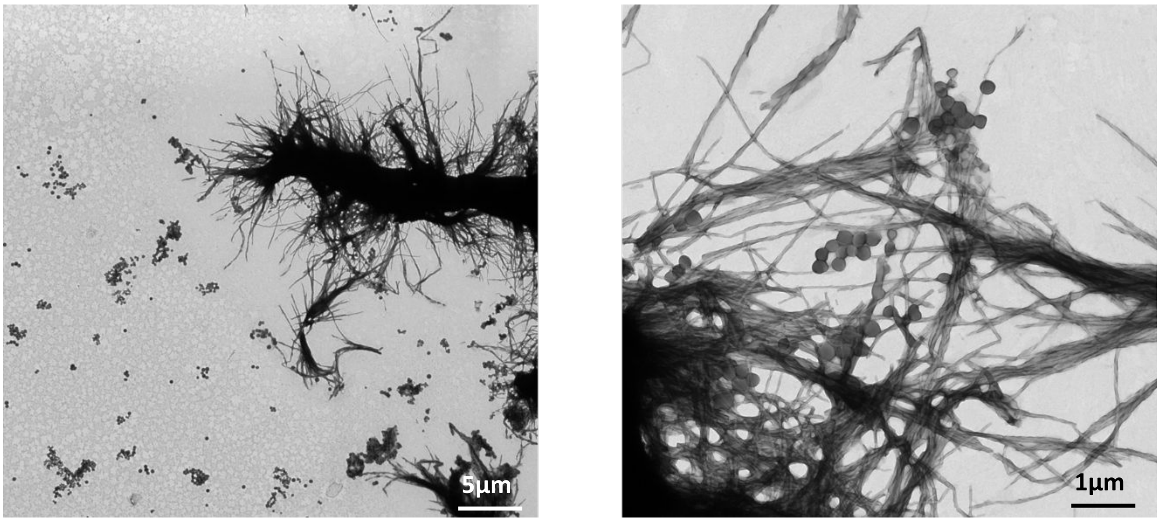

Figure S15. TEM images of organogel **2** (5 mg/mL) after ytterbium(III) chloride addition.

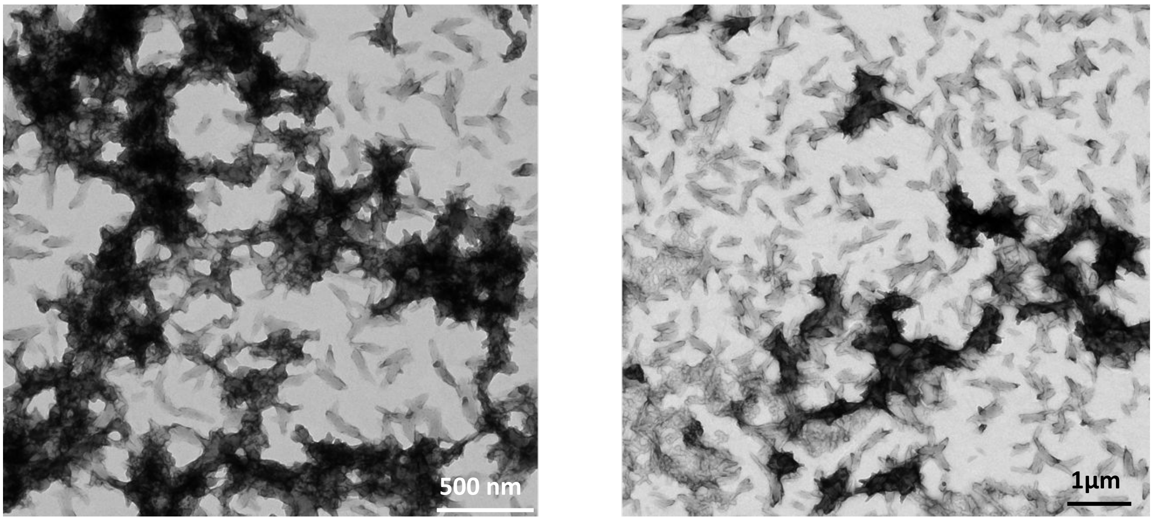

Figure S16. TEM images of organogel **2** (5 mg/mL) after europium(III) chloride addition.

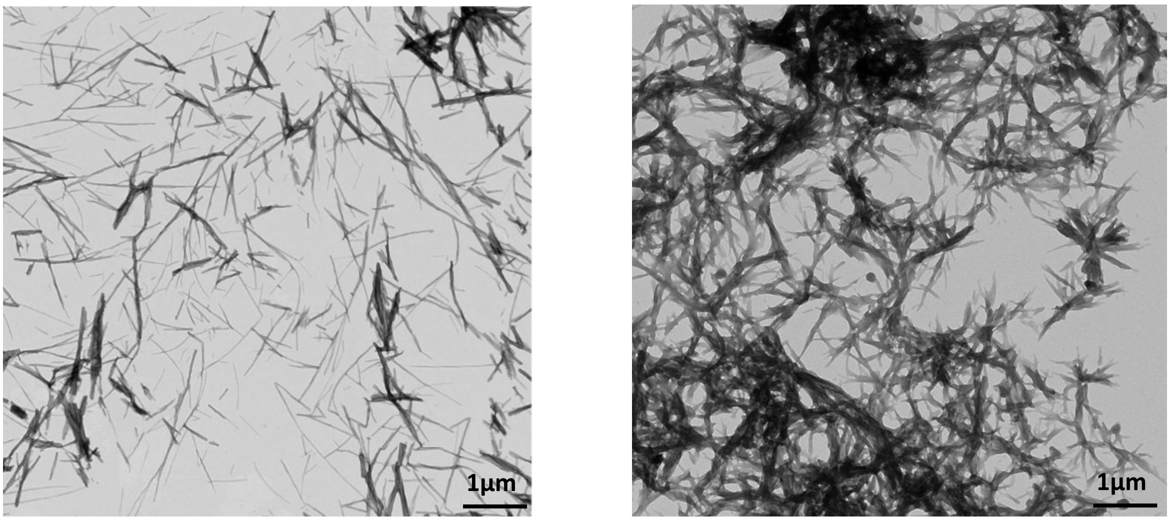

Figure S17. TEM images of organogel **2** (5 mg/mL) with the addition of sodium chlorine before (left) and after (right) the addition of potassium tetrachloroaurate(III).

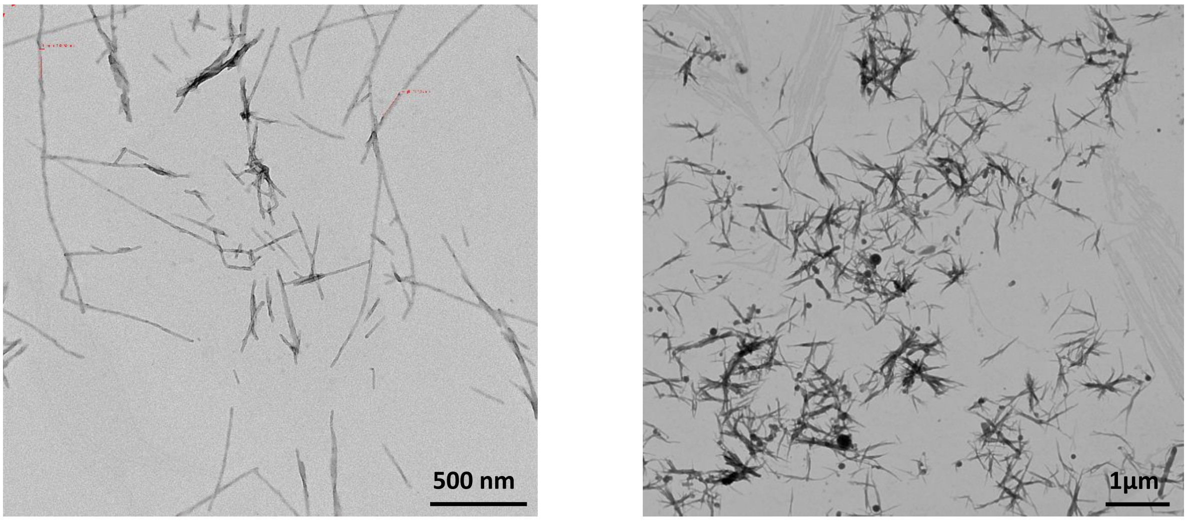

Figure S18. TEM images of organogel **2** (5 mg/mL) with the addition of calcium chloride before (left) and after (right) the addition of potassium tetrachloroaurate(III).

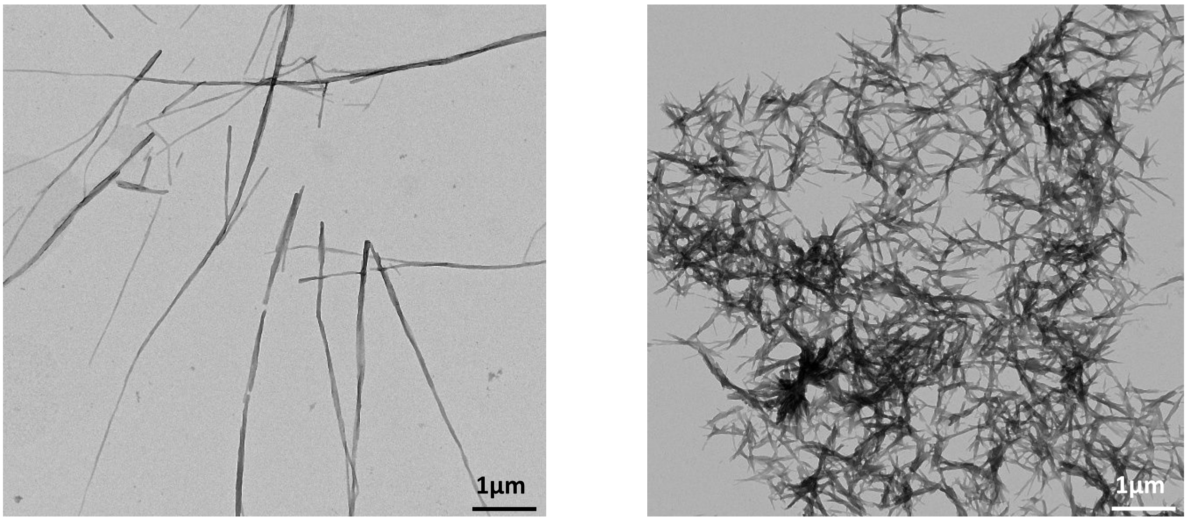

Figure S19. TEM images of organogel **2** (5 mg/mL) with the addition of copper(II) chloride dihydrate before (left) and after (right) the addition of potassium tetrachloroaurate(III).

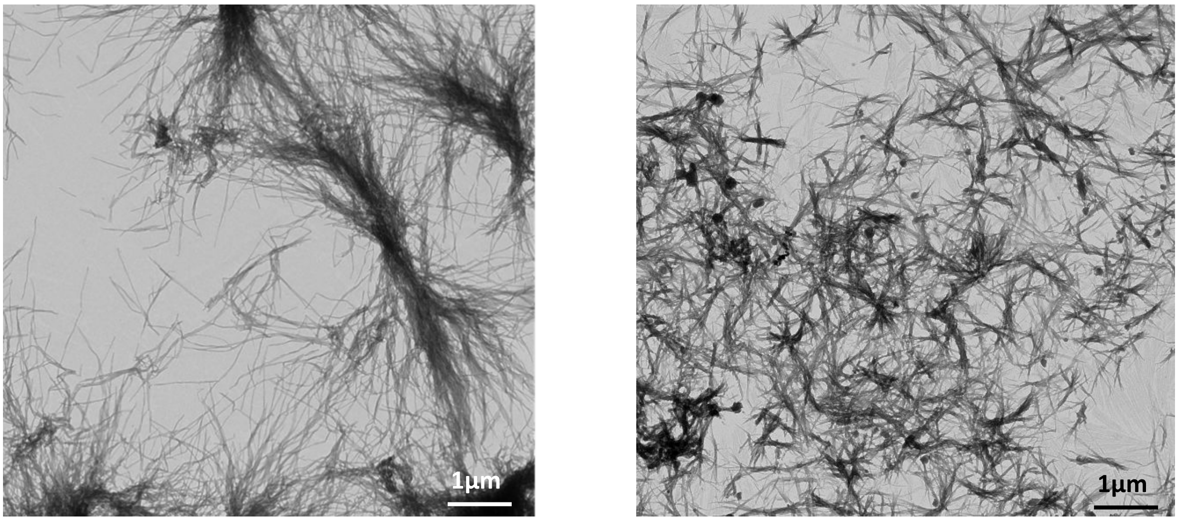

Figure S20. TEM images of organogel **2** (5 mg/mL) with the addition of silver nitrate before (left) and after (right) the addition of potassium tetrachloroaurate(III).

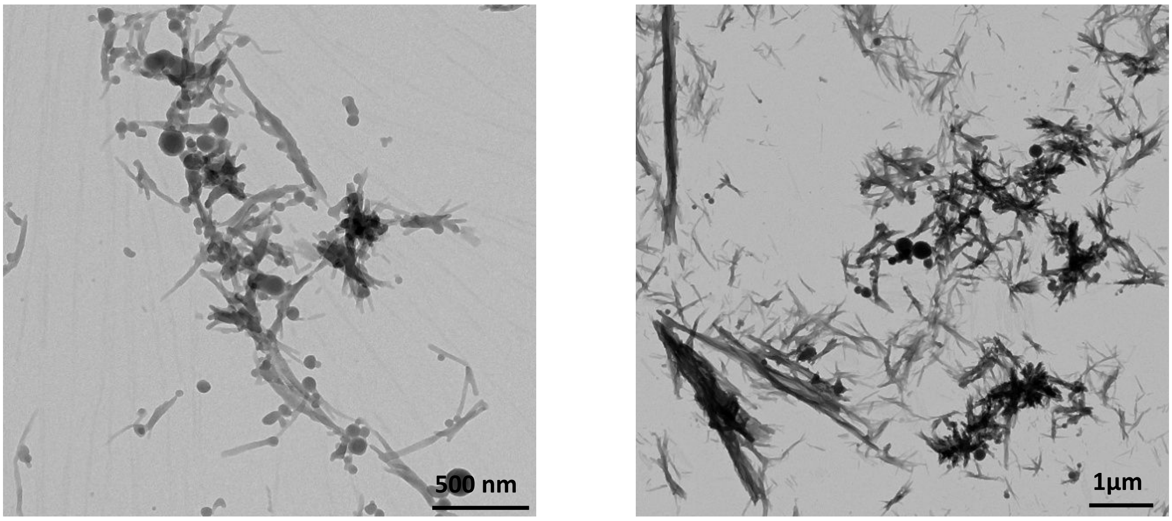

Figure S21. TEM images of organogel **2** (5 mg/mL) with the addition of palladium (II) chloride before (left) and after (right) the addition of potassium tetrachloroaurate(III).

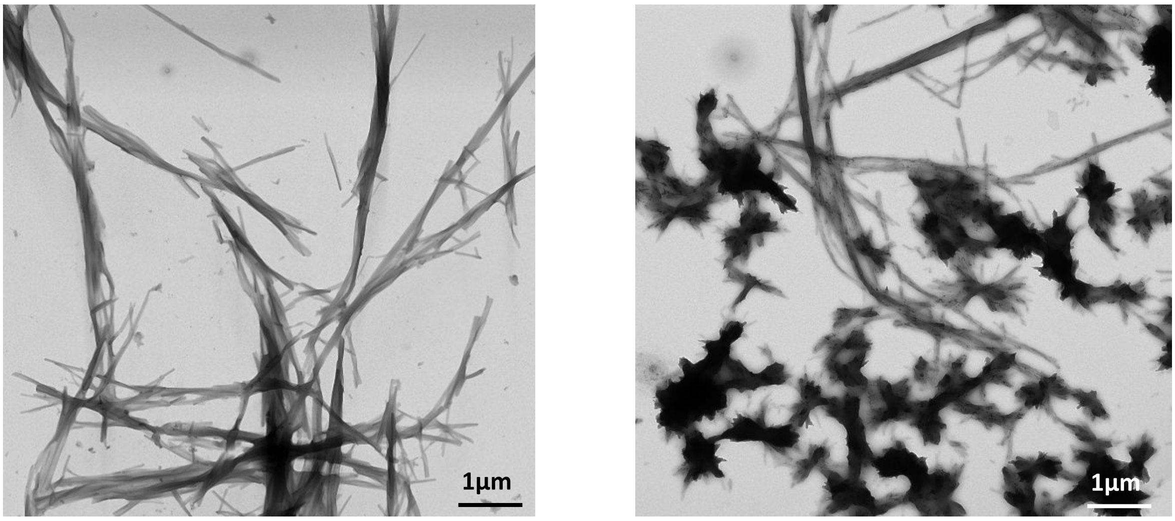

Figure S22. TEM images of organogel **2** (5 mg/mL) with the addition of chromium(III) chloride hexahydrate before (left) and after (right) the addition of potassium tetrachloroaurate(III).

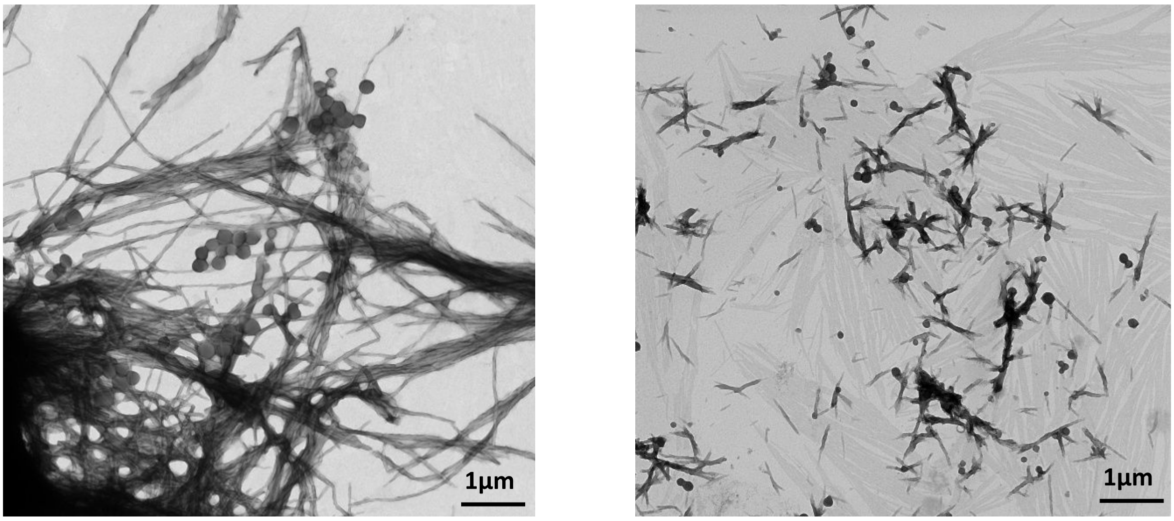

Figure S23. TEM images of organogel **2** (5 mg/mL) with the addition of ytterbium(III) chloride before (left) and after (right) the addition of potassium tetrachloroaurate(III).

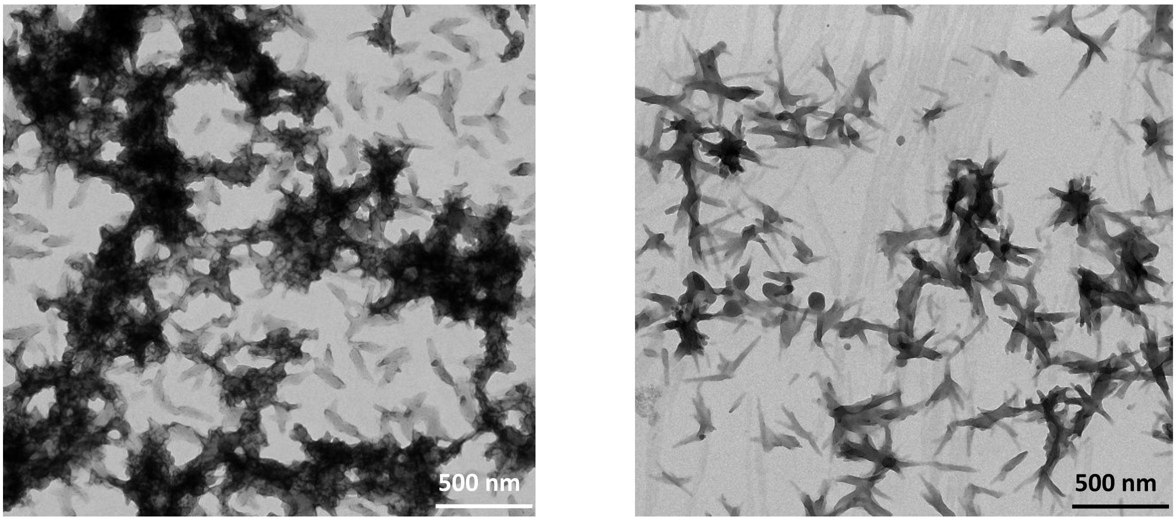

Figure S24. TEM images of organogel **2** (5 mg/mL) with the addition of europium(III) chloride before (left) and after (right) the addition of potassium tetrachloroaurate(III).

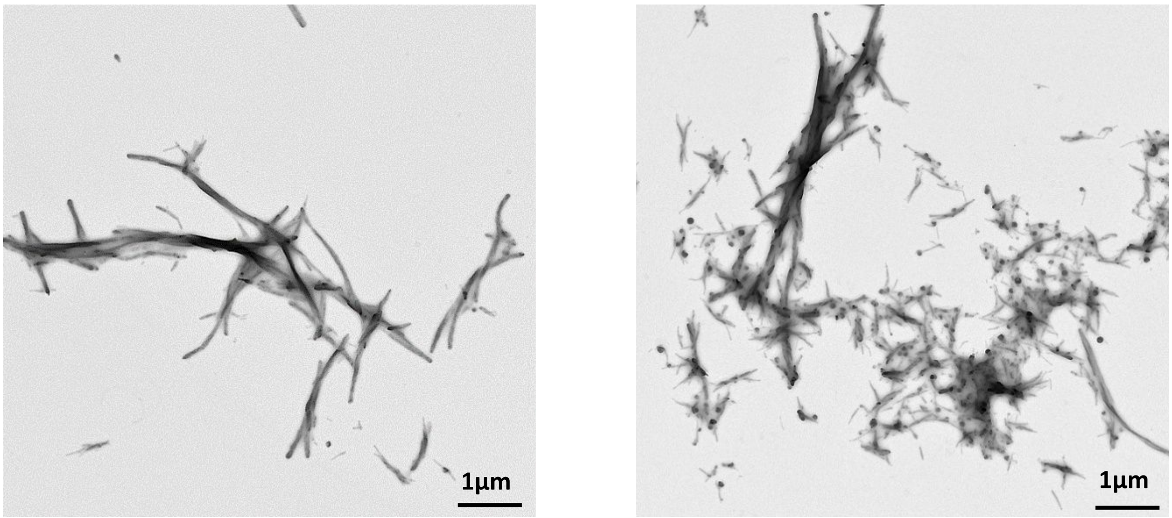

Figure S25. TEM images of the mixed organogel between **2** (5 mg/mL) and cysteine after the addition of potassium tetrachloroaurate(III).

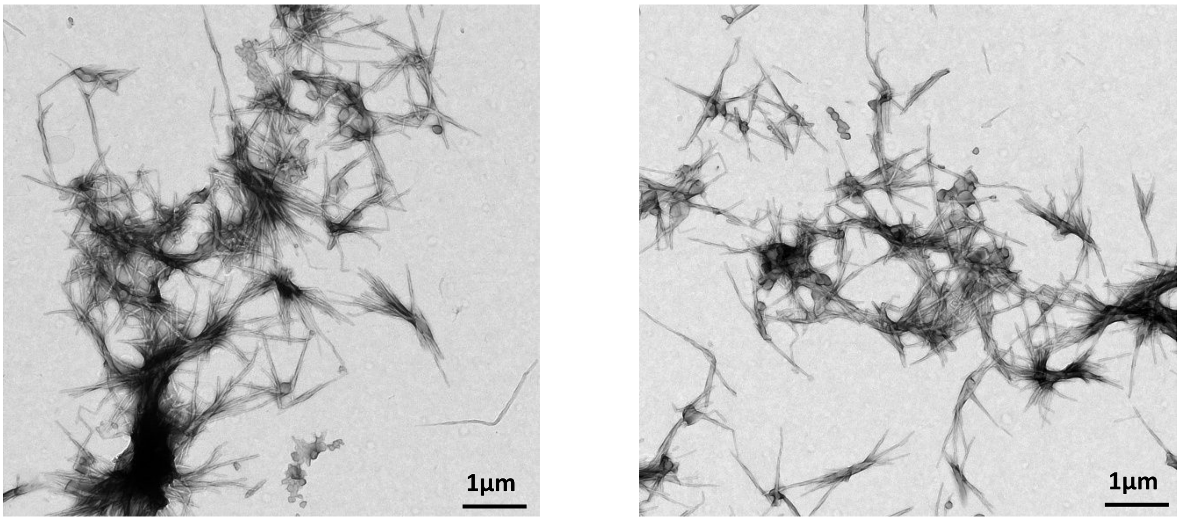

Figure S26. TEM images of organogel **2** (5 mg/mL), after the addition of potassium tetrachloroaurate(III) and the subsequent addition of cysteine.

^{31}P NMR Experiment

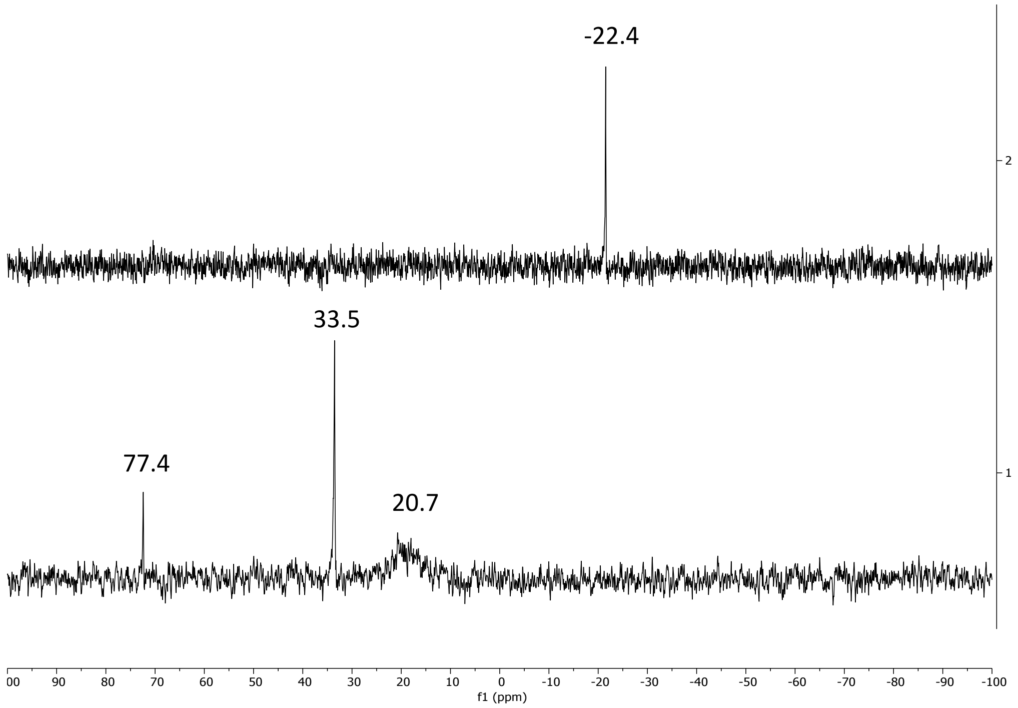

Figure S27. ^{31}P NMR spectra of compound 2 (top) and after addition of 0.1 equivalents of Au^{3+} (bottom), showing the disappearance of the free phosphine signal and the appearance of new phosphorus environments.

NMR spectra for squaramides 1-6

Squaramide 1

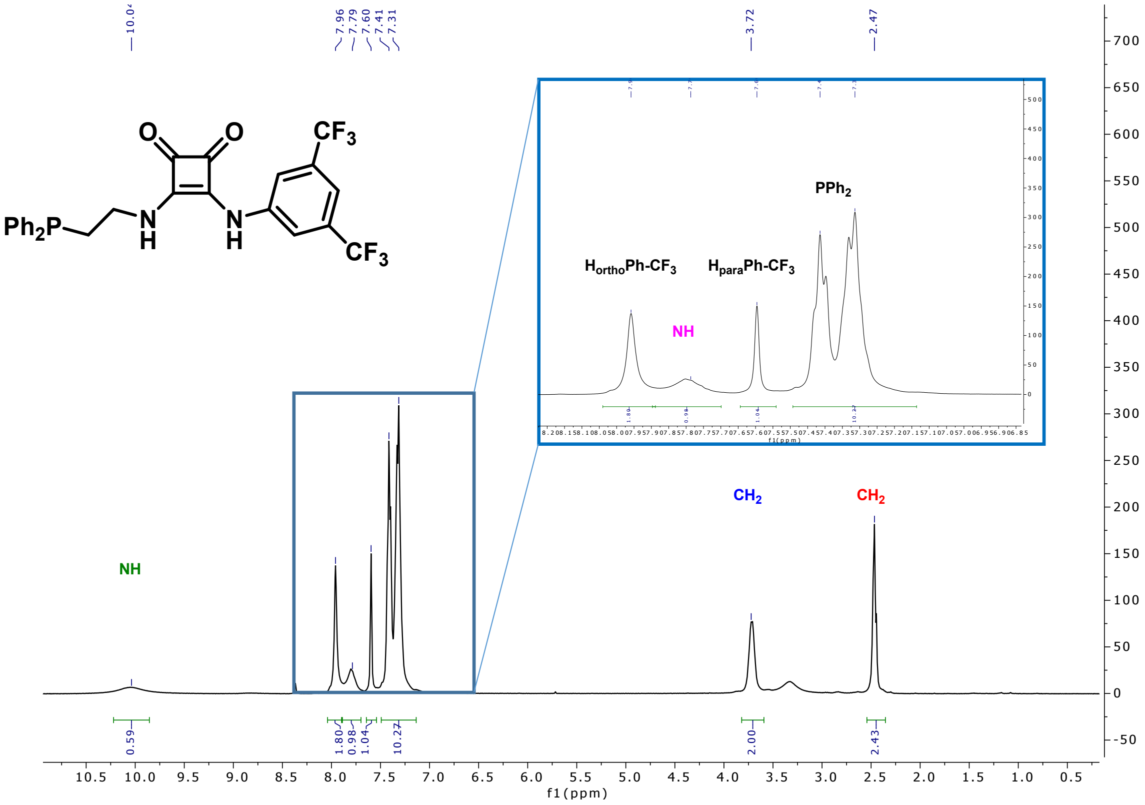

Figure S28. ^{1}H NMR (400 MHz, DMSO-d_6) spectrum of compound **1**.

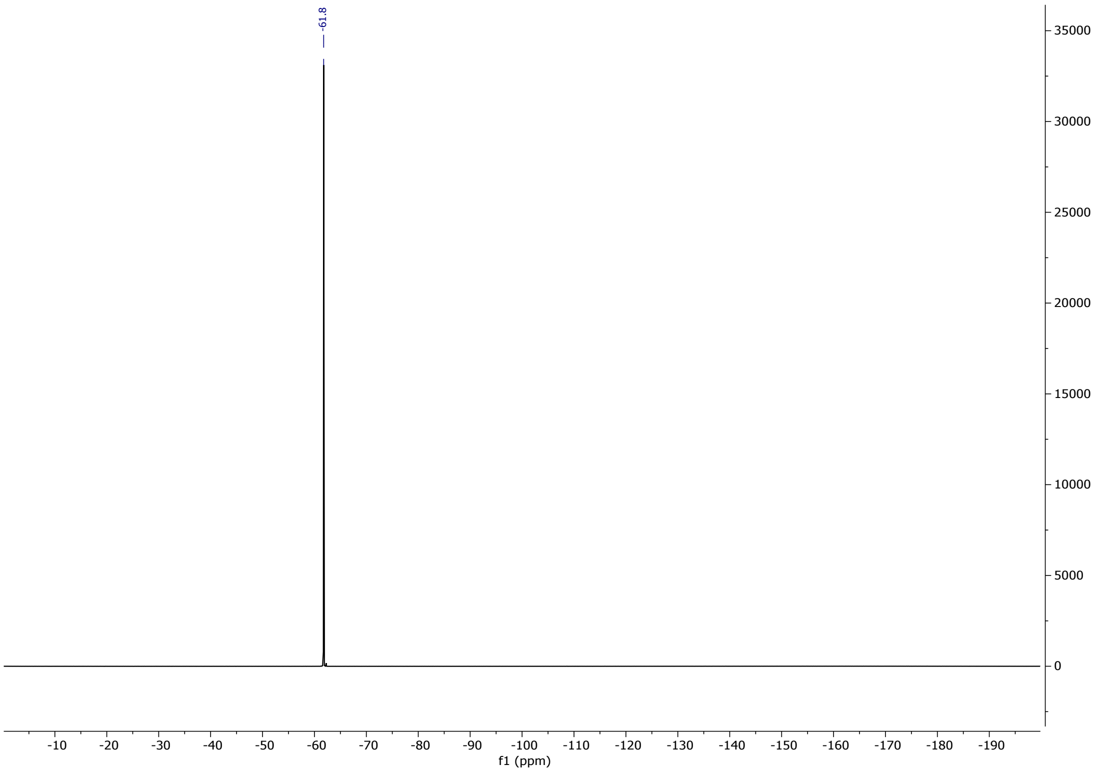

Figure S29. $^{19}F\{^{1}H\}$ NMR (ppm) (376 MHz, DMSO-d_6) spectrum of compound **1**.

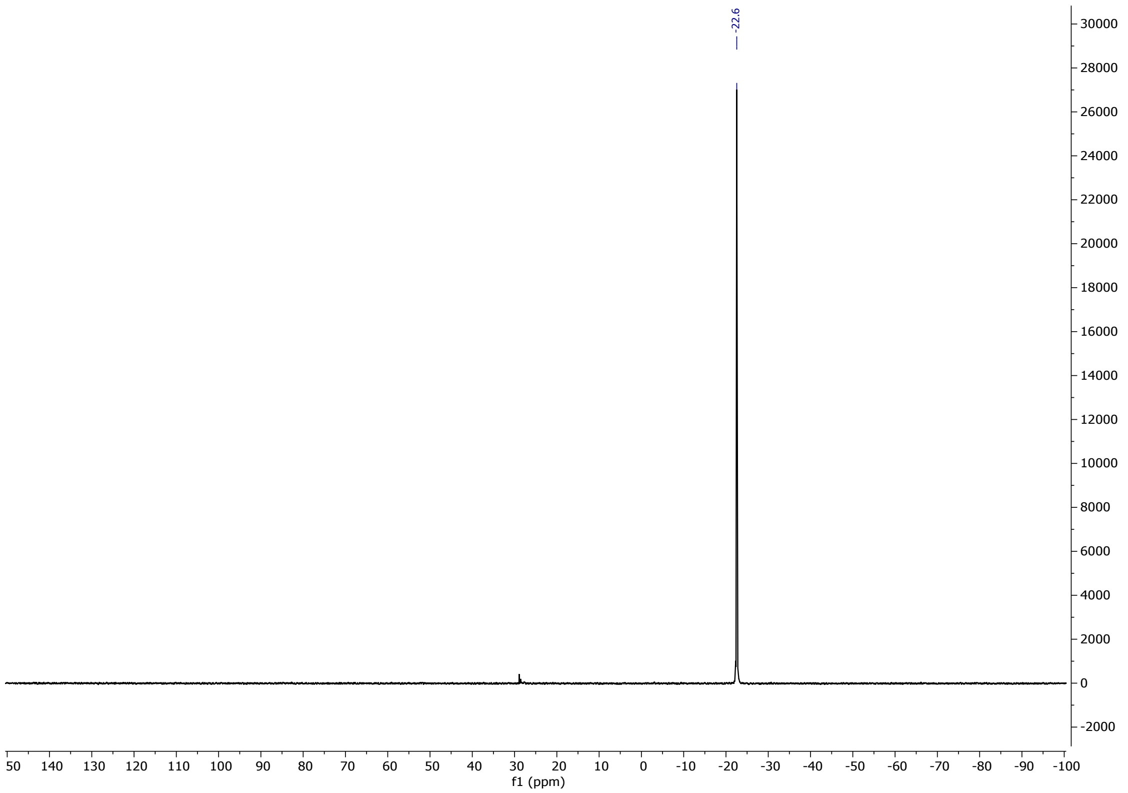

Figure S30. $^{31}P\{^{1}H\}$ NMR (ppm) (162 MHz, DMSO-d_6) spectrum of compound **1**.

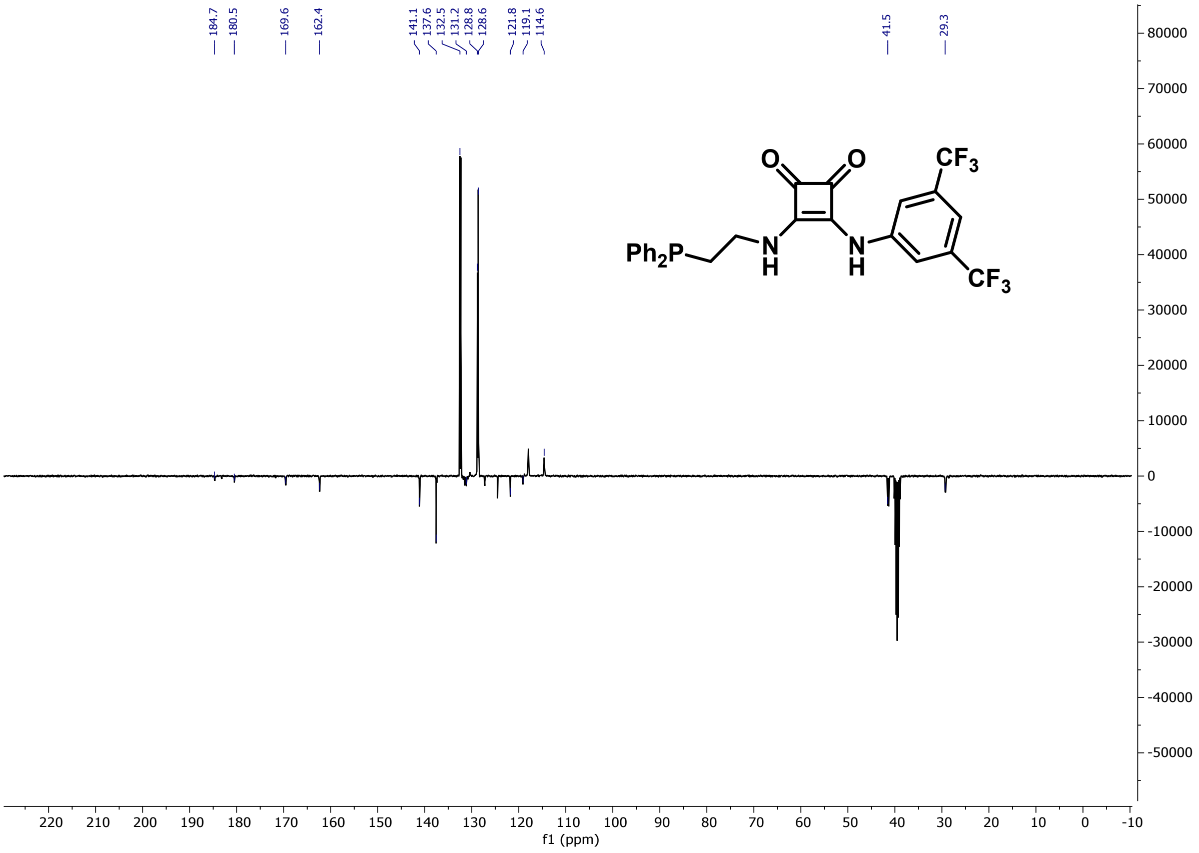

Figure S31. $^{13}C\{^{1}H\}$-APT (ppm) (100 MHz, DMSO-d_6) spectrum of compound **1**.

Squaramide 2

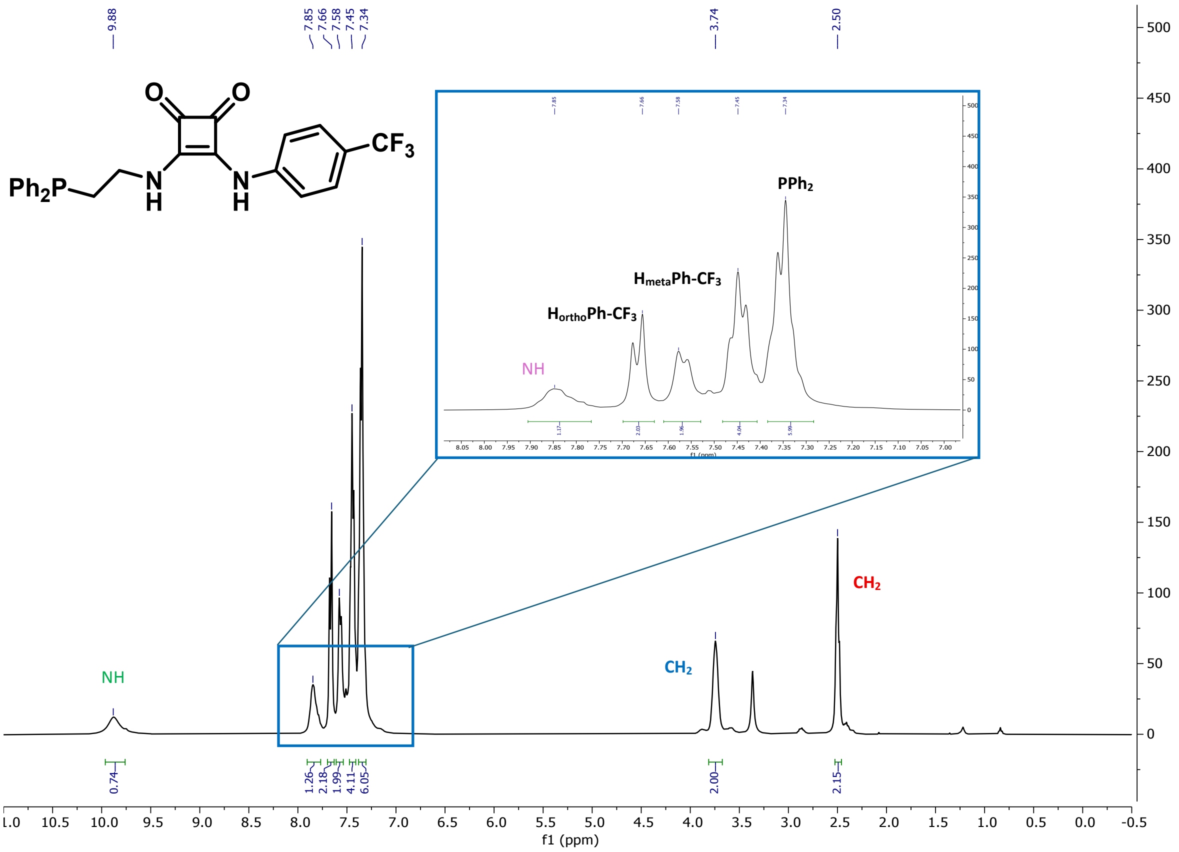

Figure S32. ^{1}H NMR (ppm) (400 MHz, DMSO-d_6) spectrum of compound **2**.

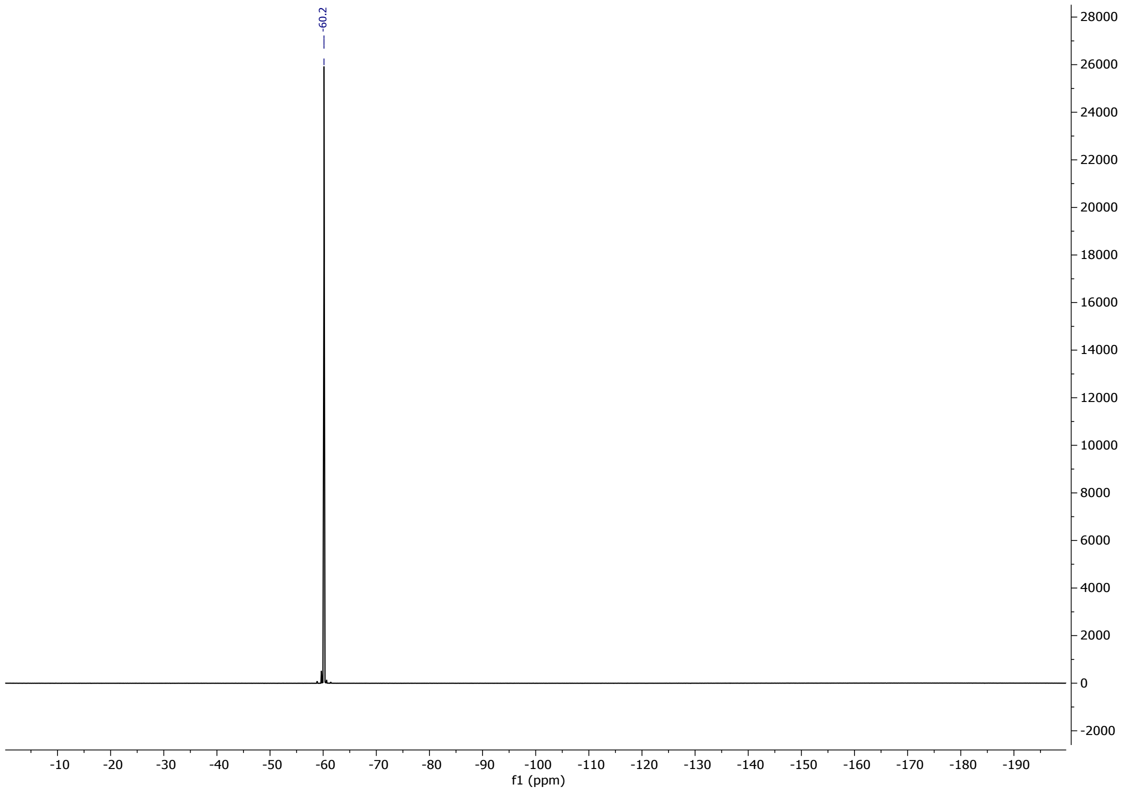

Figure S33. $^{19}F\{^{1}H\}$ NMR (ppm) (376 MHz, DMSO-d_6) spectrum of compound **2**.

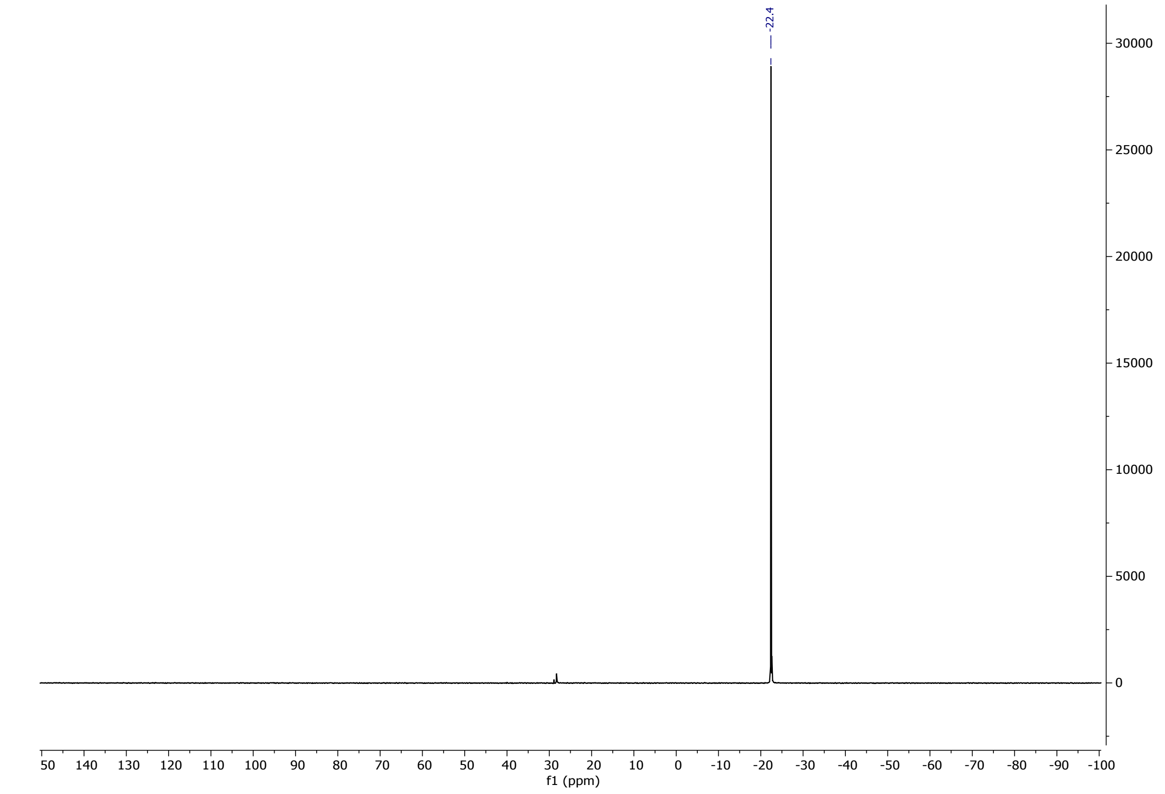

Figure S34 $^{31}P\{^{1}H\}$ NMR (ppm) (162 MHz, DMSO-d_6) spectrum of compound **2**.

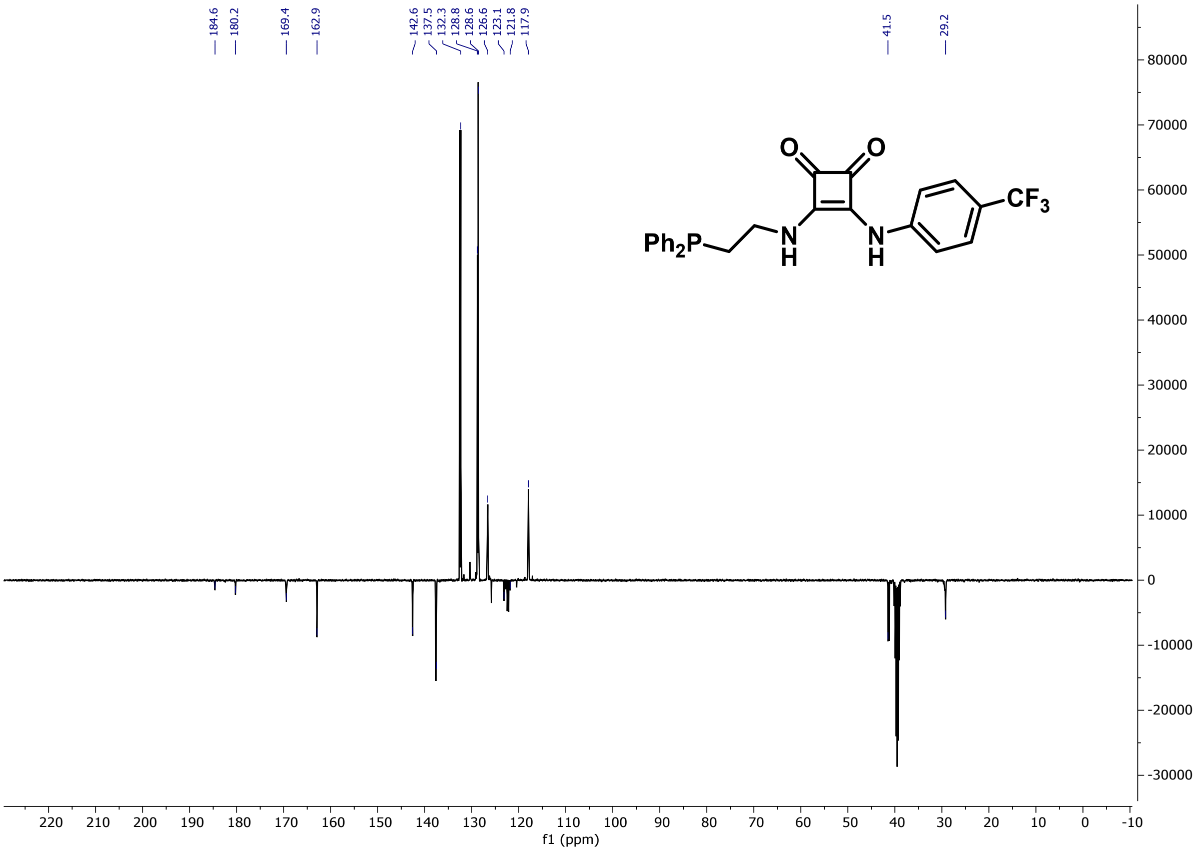

Figure S35. $^{13}C\{^{1}H\}$-APT (ppm) (100 MHz, DMSO-d_6) spectrum of compound **2**.

Squaramide 3

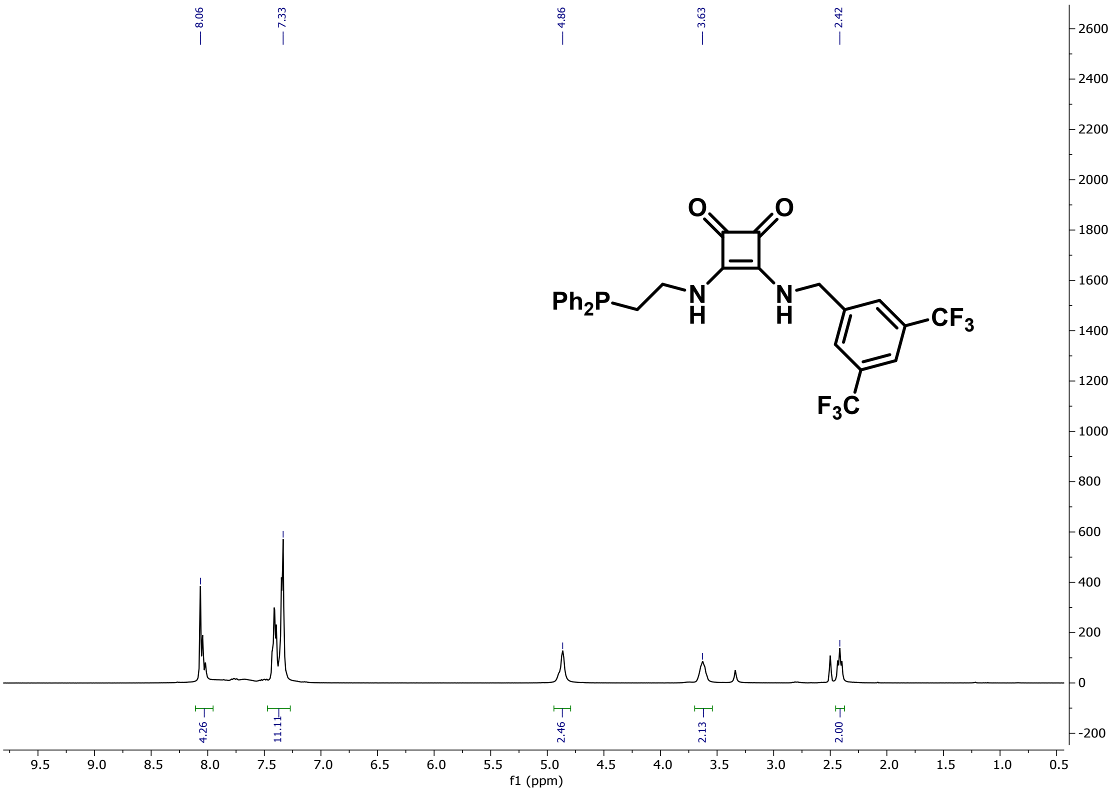

Figure S36. ^{1}H NMR (ppm) (400 MHz, DMSO-d_6) spectrum of compound **3**.

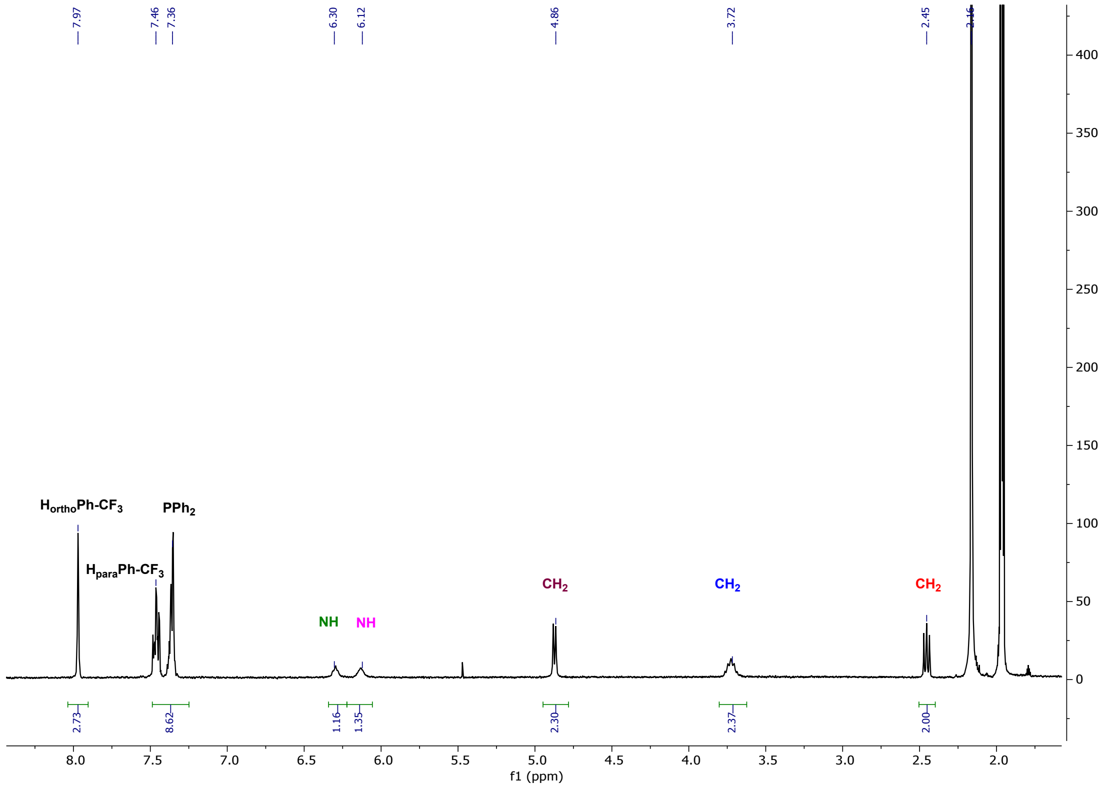

Figure S37. ^{1}H NMR (ppm) (400 MHz, CD_3CN) spectrum of compound **3**.

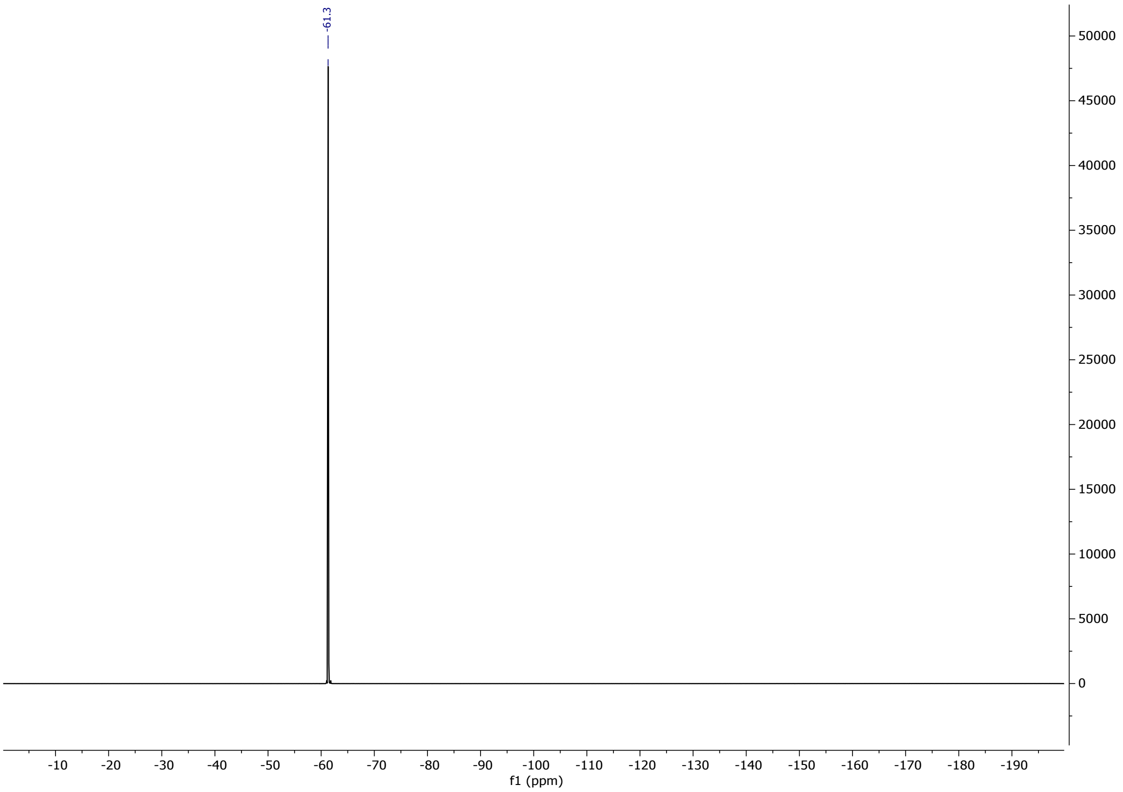

Figure S38. $^{19}F\{^{1}H\}$ NMR (ppm) (376 MHz, DMSO-d_6) spectrum of compound **3**.

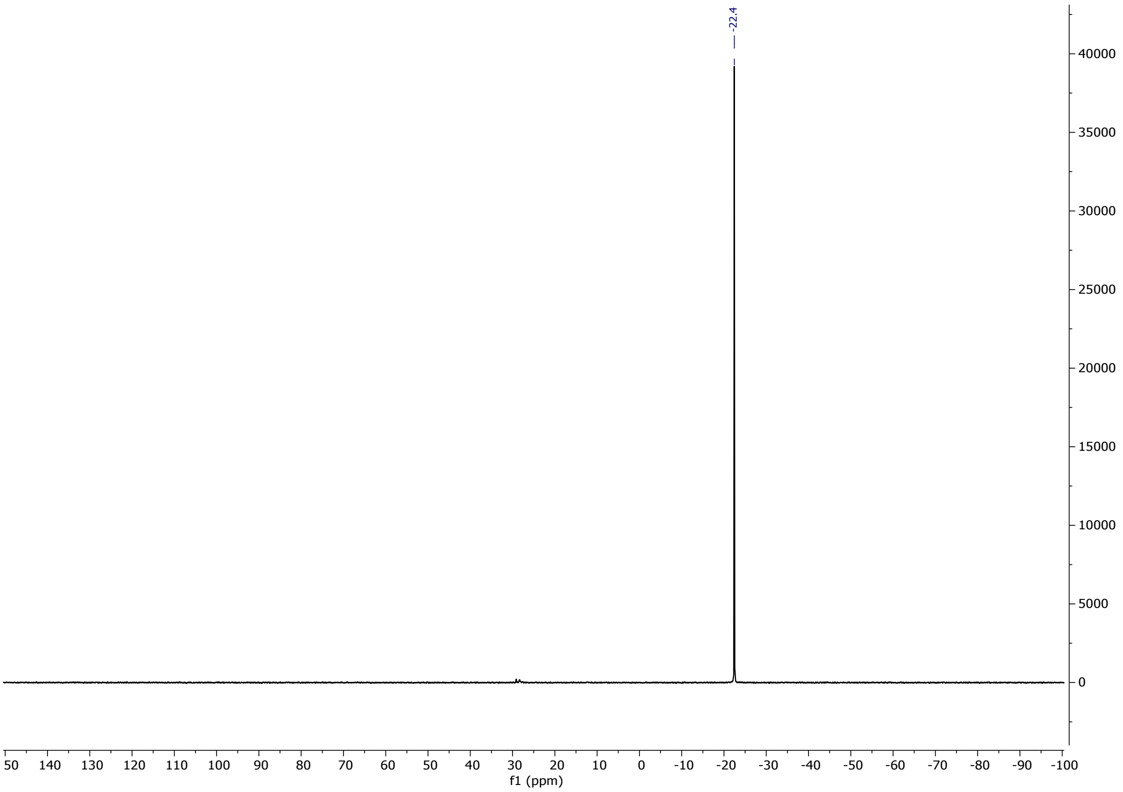

Figure S39. $^{31}P\{^{1}H\}$ NMR (ppm) (162 MHz, DMSO-d_6) spectrum of compound **3**.

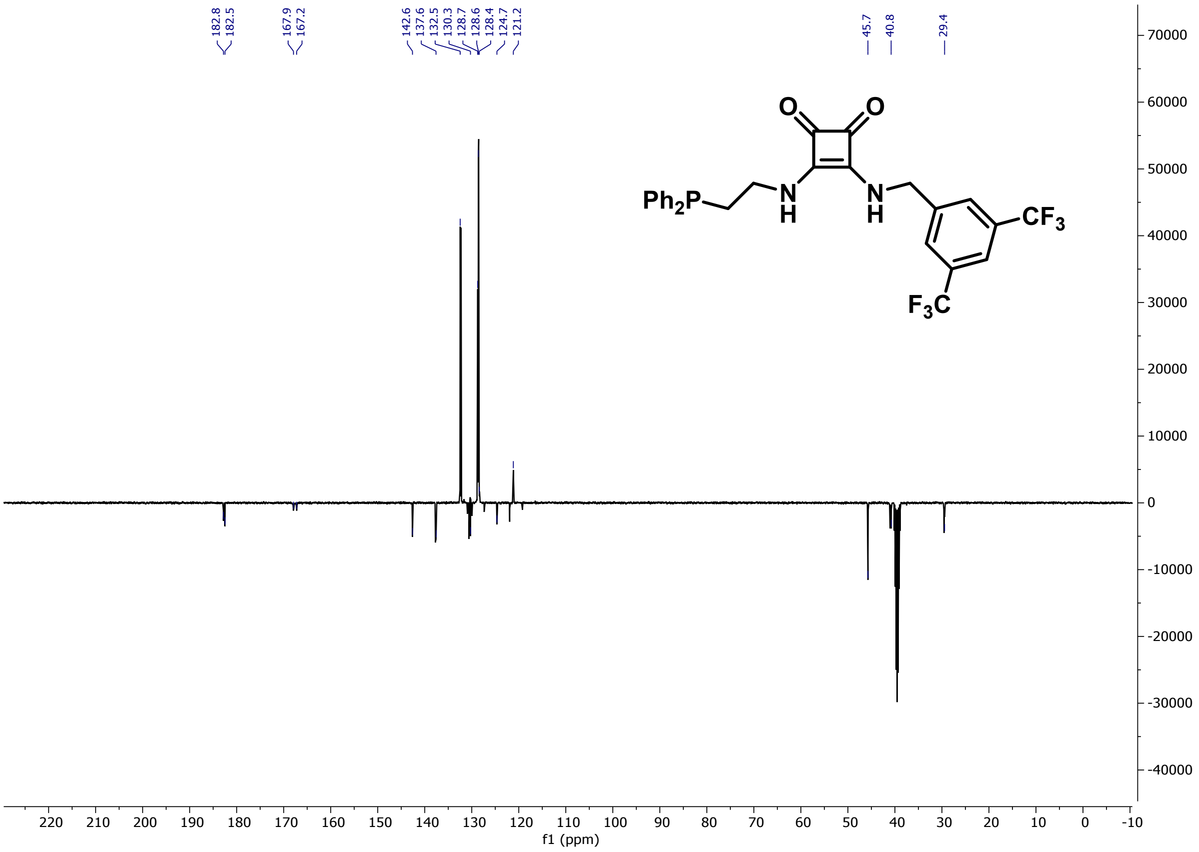

Figure S40. $^{13}C\{^{1}H\}$-APT spectrum of compound **3** in DMSO-d_6 solution.

Squaramide 4

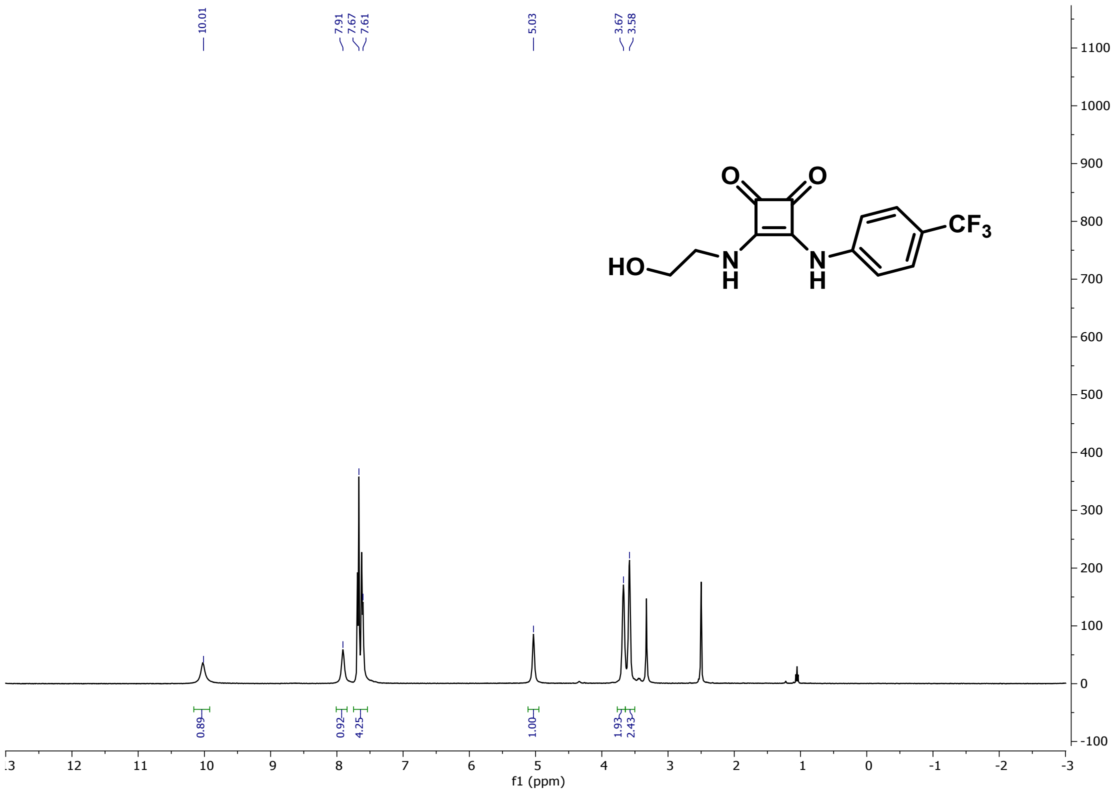

Figure S41. ^{1}H NMR (ppm) (400 MHz, DMSO-d_6) spectrum of compound **4**.

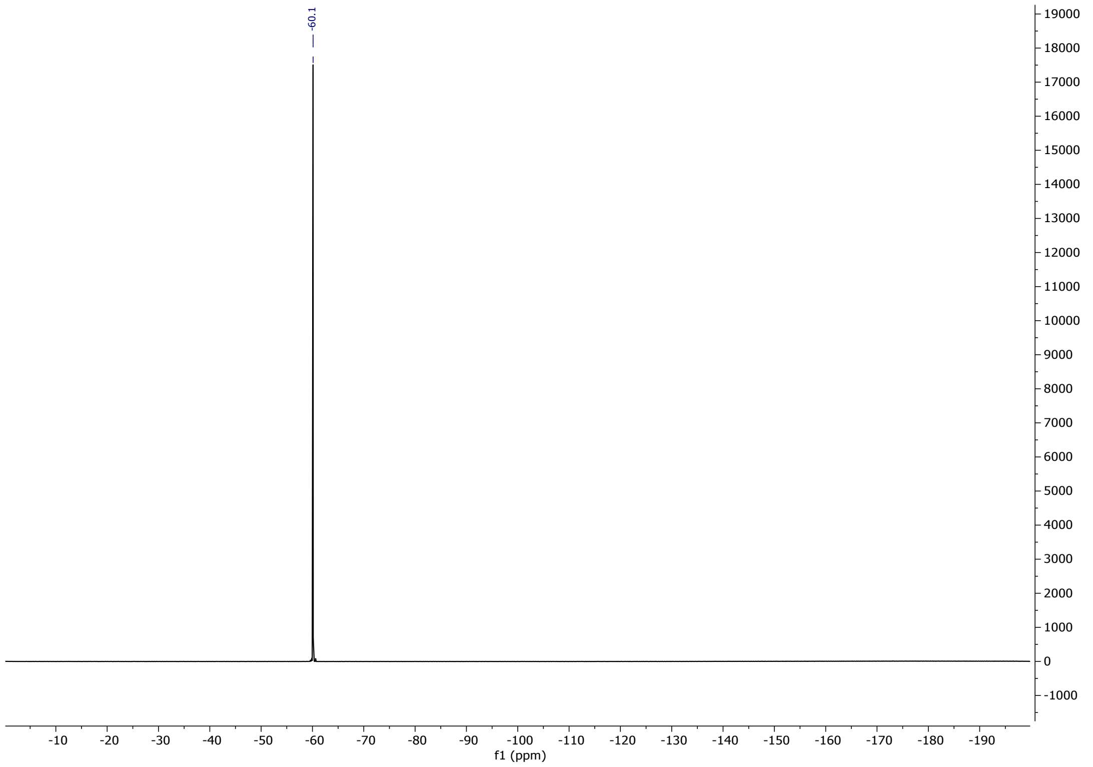

Figure S42. $^{19}F\{^{1}H\}$ NMR (ppm) (376 MHz, DMSO-d_6) spectrum of compound **4**.

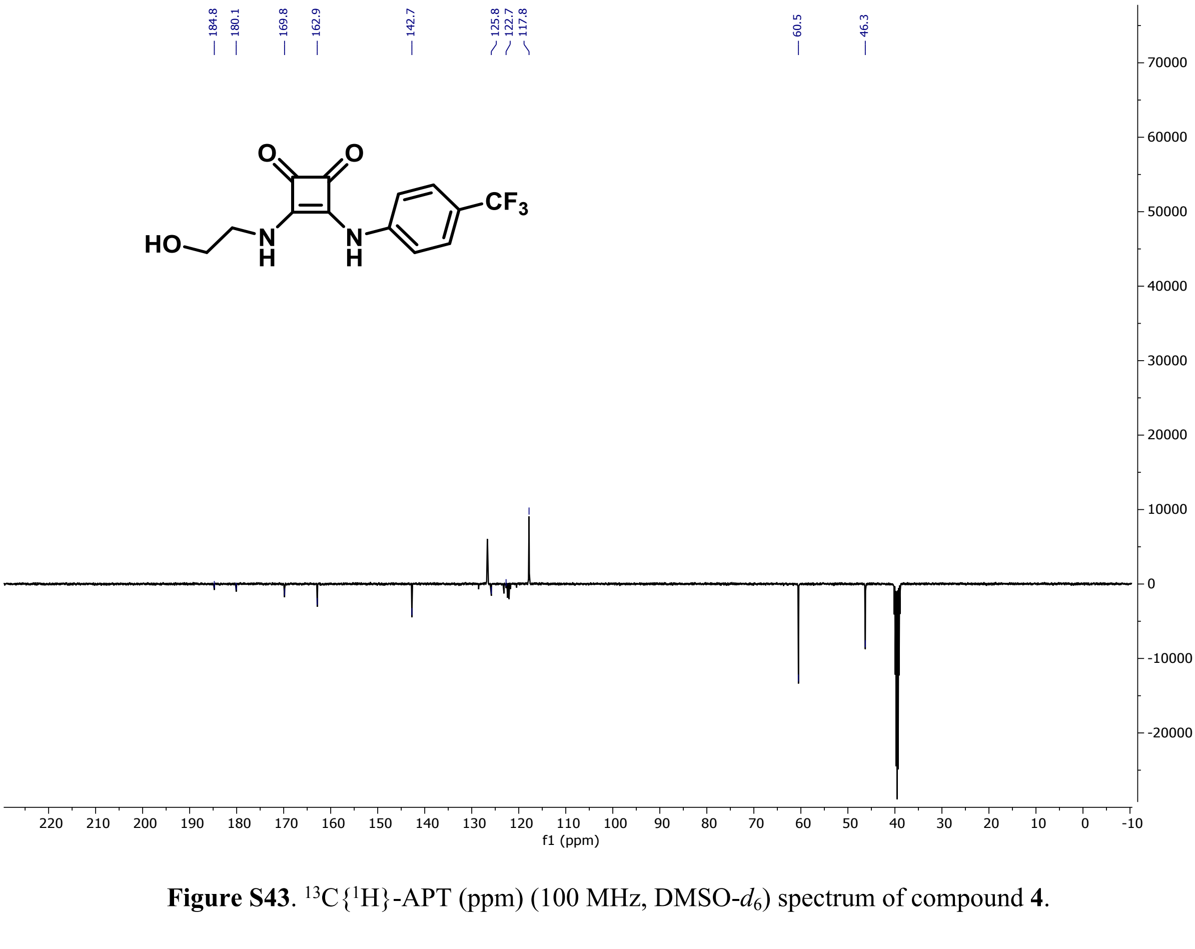

Figure S43. $^{13}C\{^{1}H\}$-APT (ppm) (100 MHz, DMSO-d_6) spectrum of compound **4**.

Squaramide 5

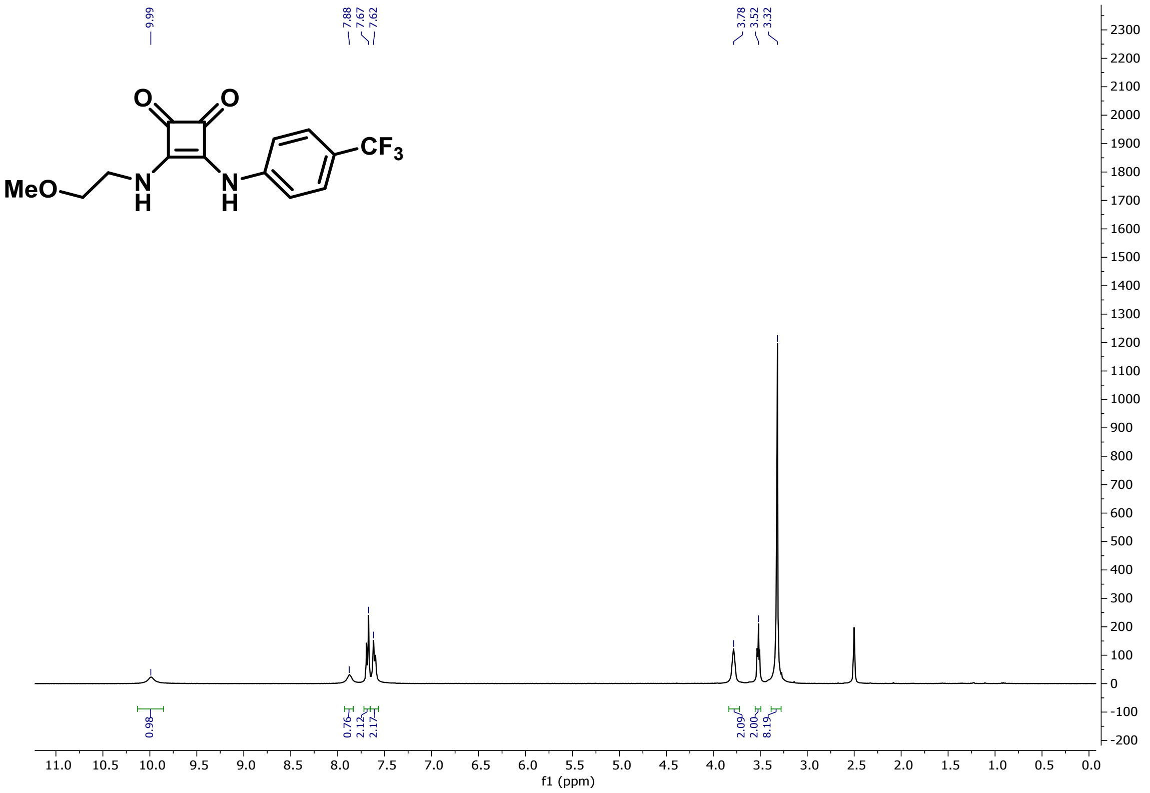

Figure S44. ^{1}H NMR (ppm) (400 MHz, DMSO-d_6) spectrum of compound **5**.

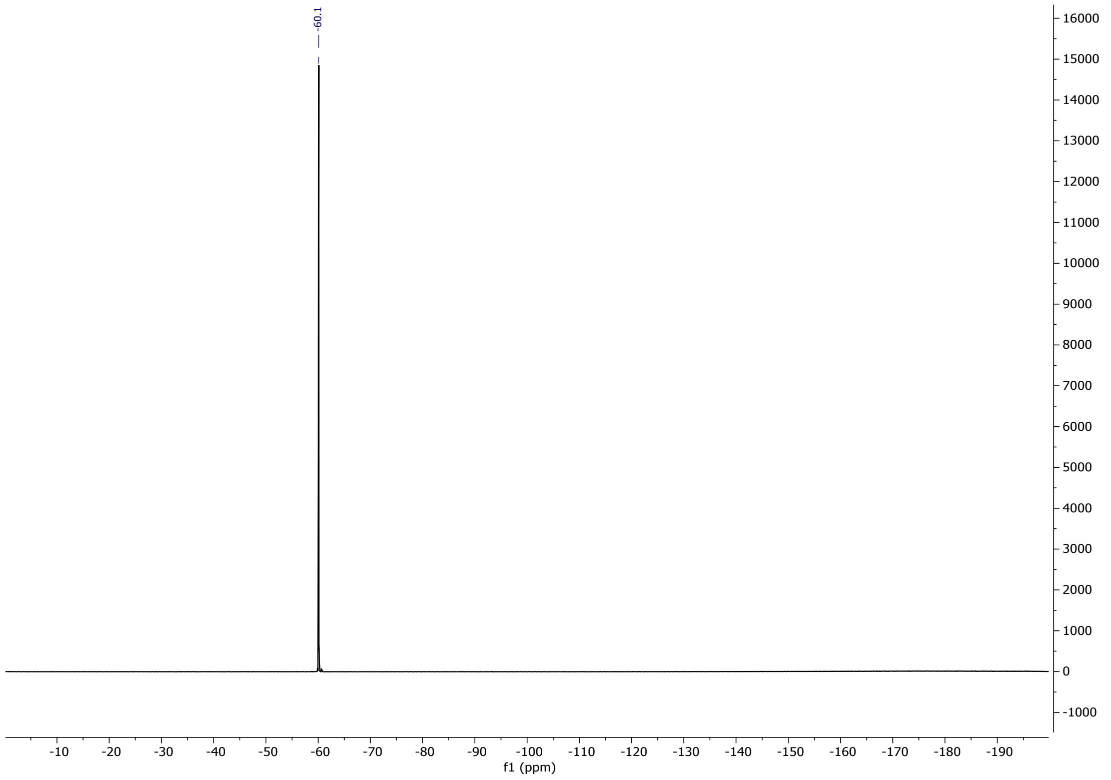

Figure 45. ^{19}F{^{1}H} NMR (ppm) (376 MHz, DMSO-d_6) spectrum of compound **5**.

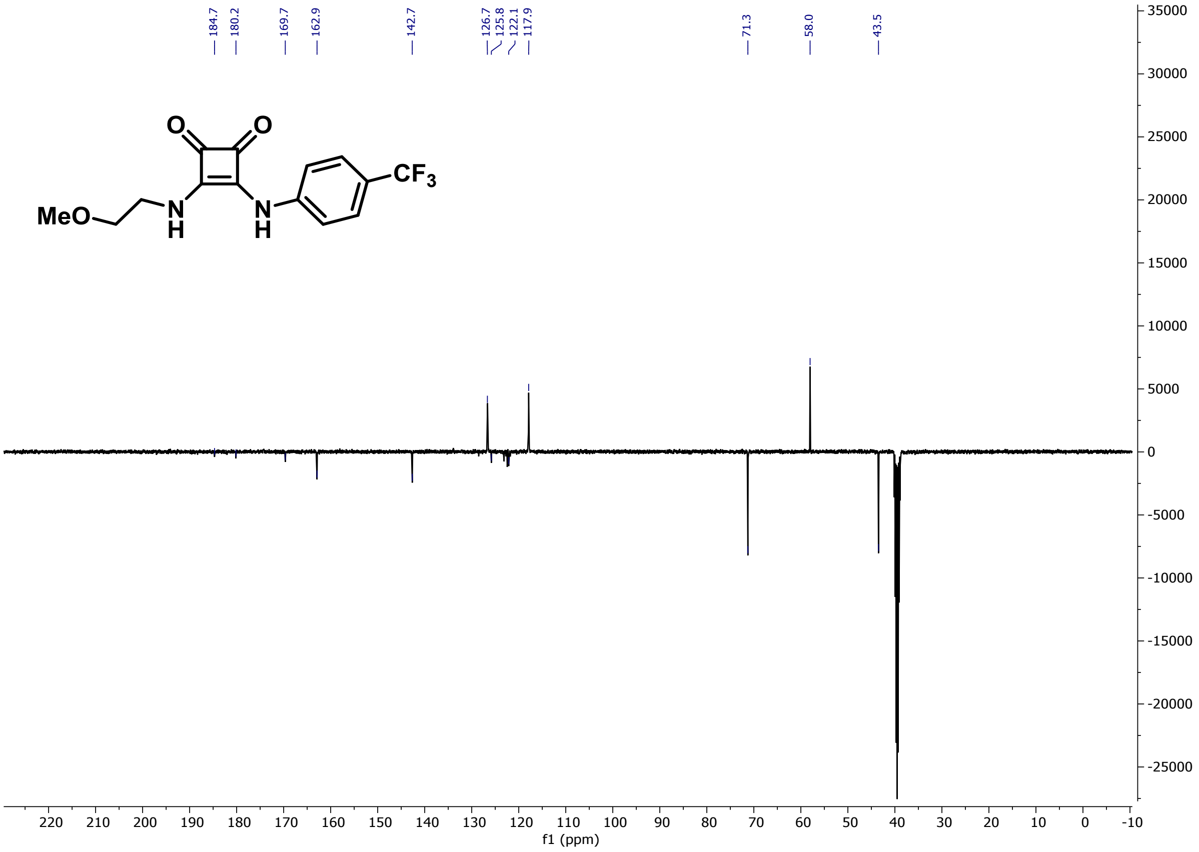

Figure S46. $^{13}C\{^{1}H\}$-APT (ppm) (100 MHz, DMSO-d_6) spectrum of compound **5**.

Squaramide 6

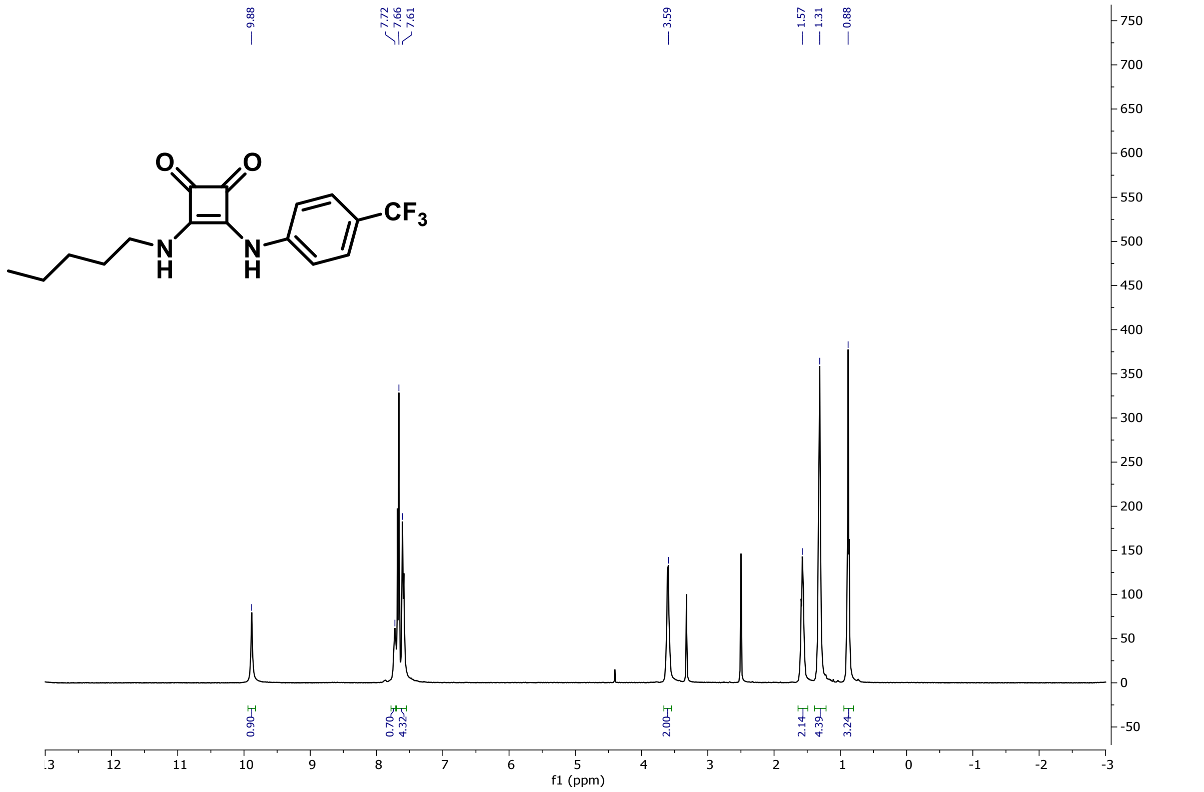

Figure S47. ^{1}H NMR (ppm) (400 MHz, DMSO-d_6) spectrum of compound **6**.

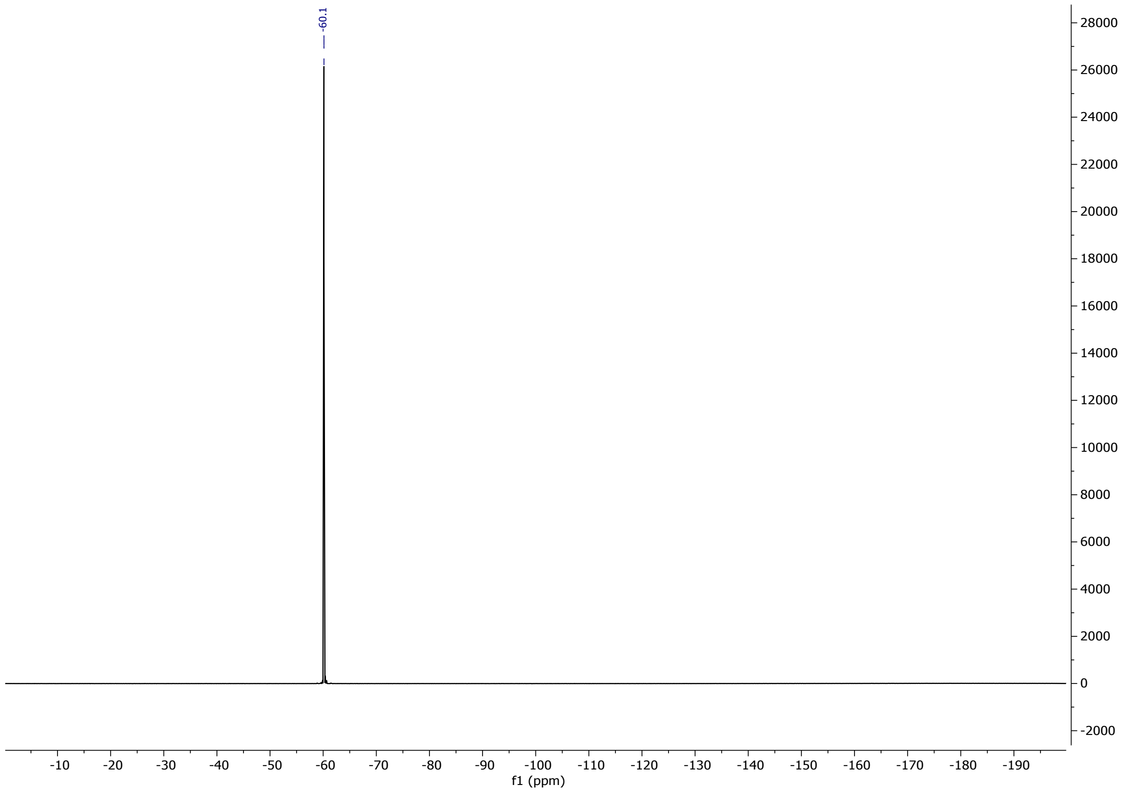

Figure S48. $^{19}F\{^{1}H\}$ NMR (ppm) (376 MHz, DMSO-d_6) spectrum of compound **6**.

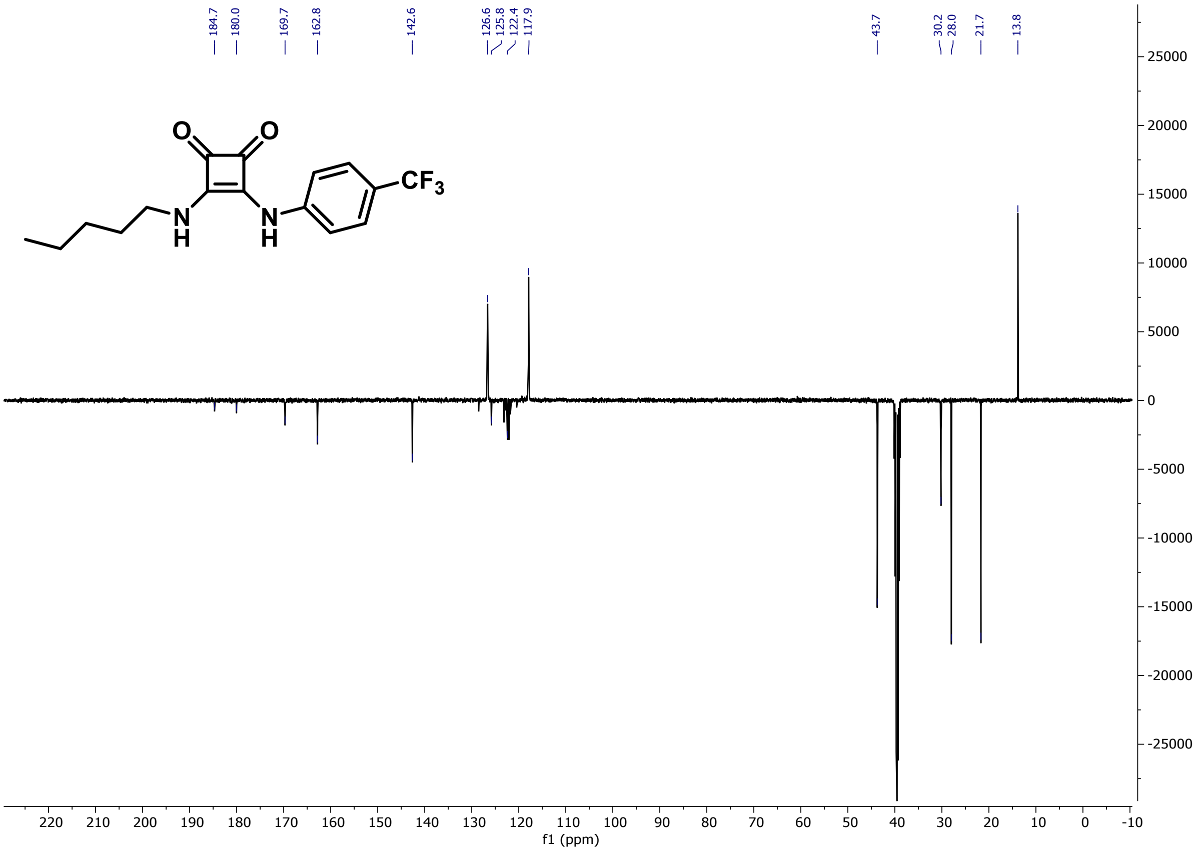

Figure S49. $^{13}C\{^{1}H\}$-APT (ppm) (100 MHz, DMSO-d_6) spectrum of compound **6**.
